# IGZO‐Based First Spike Timing Tactile Encoders and Coupling‐Enhanced Transistor Synapses for Efficient Spiking Neural Networks

**DOI:** 10.1002/advs.202511168

**Published:** 2025-12-08

**Authors:** Dan Cai, Jinyong Wang, Tianchen Zhao, Miao Shen, Yunbo Liu, Tieyi Zhang, Fangjie Zhang, Yang Wang, Yadong Jiang, Deen Gu

**Affiliations:** ^1^ School of Optoelectronic Science and Engineering University of Electronic Science and Technology of China Chengdu 611731 P. R. China; ^2^ State Key Laboratory of Electronic Thin Films and Integrated Devices University of Electronic Science and Technology of China Chengdu 610054 P. R. China; ^3^ Department of Electrical and Computer Engineering National University of Singapore Singapore 117583 Singapore; ^4^ Mianyang Huike Optoelectronic Technology Co. Ltd. Mianyang 621000 P. R. China

**Keywords:** coupling‐enhanced, first spike timing, IGZO, SNN, synapse

## Abstract

Spike encoding is the fundamental prerequisite for the hardware implementation of event‐driven spiking neural networks (SNNs). However, compact device‐level realization of first‐spike‐timing (FST) encoding remains challenging, while high‐performance synaptic devices are urgently needed for efficient network training. Here, a light‐accelerated SNN hardware framework is proposed that integrates sensing, temporal encoding, and synaptic learning. A PDMS/MWCNTs film with IGZO dual‐TFTs (PDTFT) enables precise subthreshold modulation to restore the neuron “resting state,” achieving millisecond‐scale FST tactile encoding. Meanwhile, a GaOx/IGZO heterojunction introduced as a light‐electric coupling synapse (LECTS), where light supplements carriers and electrical bias modulates the barrier, overcoming the intrinsic lack of long‐term memory in IGZO and enabling stronger plasticity beyond single stimuli. Combining PDTFT and LECTS, autonomous‐vehicle status detection (98.4% accuracy) are demonstrated and smart robotic navigation (98.2% accuracy) with a 90.9% reduction in training time under supervised SNN learning. These results demonstrate a compact and highly‐efficient strategy for neuromorphic intelligence systems.

## Graphical abstract

Here, a bioinspired light‐accelerated neuromorphic system that seamlessly links tactile sensing, first‐spike‐timing (FST) encoding, and light–electric synaptic learning. Pressure stimuli trigger FST spikes in dual‐gate PDTFTs, while GaOx/IGZO hetero‐synapses exhibit enhanced memory under optical–electrical co‐activation, enabling spiking neural networks to learn faster—with a remarkable 90.9% reduction in training time.

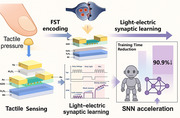

## 1 Introduction

Event‐driven spiking neural networks (SNNs) are reckoned as promising candidates for the next‐generation high‐performance neuromorphic computing systems owing to their brain‐inspired architecture and information processing paradigm, making them ideal for highly energy‐efficient intelligent decision‐making systems.^[^ ^1^, ^2^ ^]^ The prerequisite for SNN hardware implementation is encoding sensory information into sparse, spatiotemporal spike trains.^[^ ^3^, ^4^, ^5^ ^]^ Rate coding is a widely used encoding scheme for SNNs. It is extensively explored for developing intelligent systems by integrating sensors with CMOS circuits or specialized devices.^[^ ^6^, ^7^, ^8^, ^9^, ^10^ ^]^ However, rate coding causes a remarkable loss of the dynamic information from sensory inputs, resulting in the degradation of temporal resolution.^[^ ^11^ ^]^

**Figure 1:**
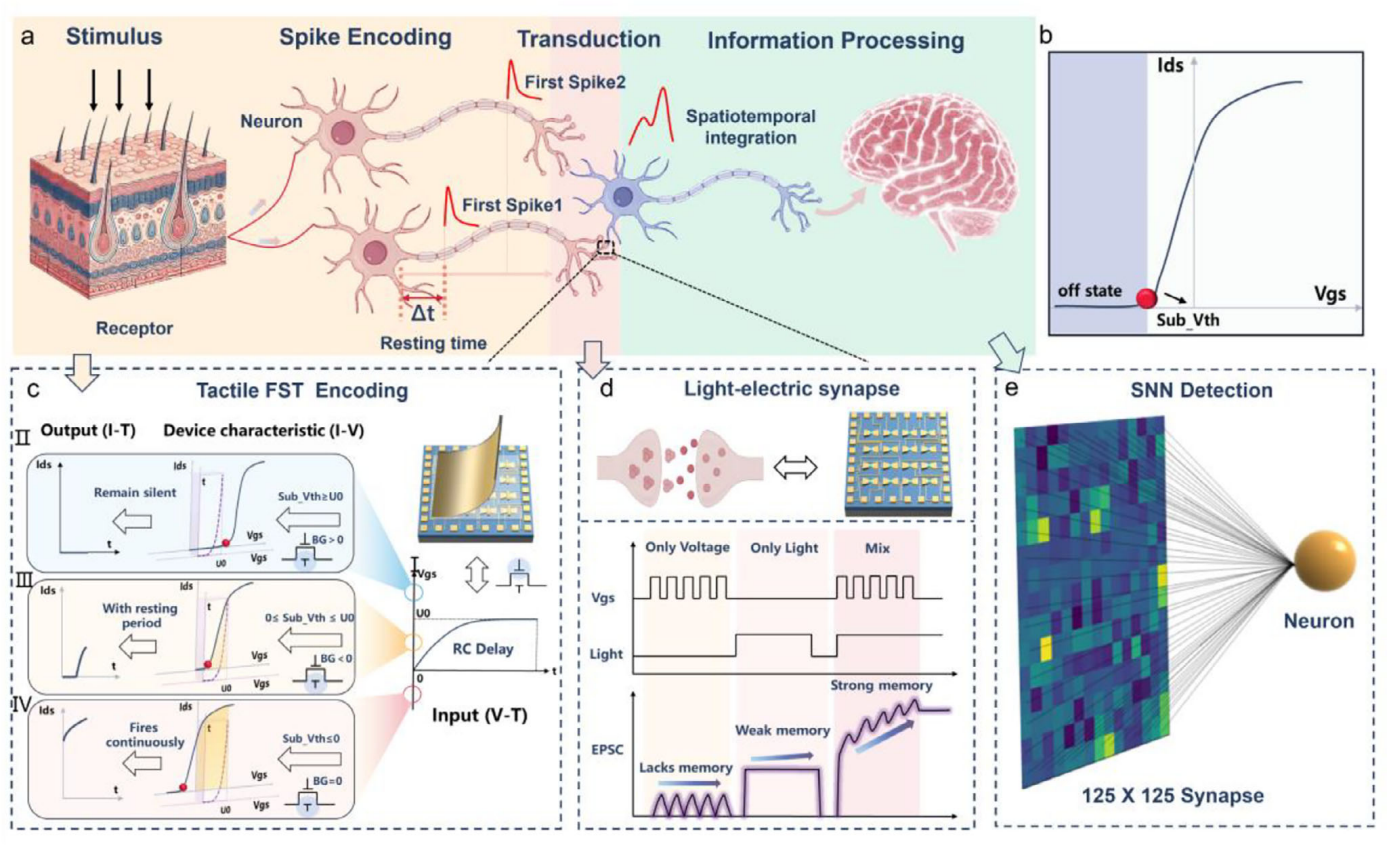
Schematic representation of biological and hardware components in SNNs. a) Schematic diagram of the tactile information processing pathway in the biological system. External stimuli activate sensory neurons, generating spike trains with FST encoding. Synaptic plasticity modulates spike amplitudes, and the integration of spike timing and amplitude differences occurs spatiotemporally in the postsynaptic neuron. b) The definition of Sub_Vth in IGZO TFTs, which represents the critical point between the off‐state and the subthreshold region in the ID‐VG curve of the device. c) Illustration of the necessity of Sub_Vth for modulation into the charging region and how BG modulation ensures Sub_Vth stays within the charging region. d) Electrical responses of LECTS under three stimulation modes: light‐only, electrical‐only, and combined light‐electrical. e) Demonstration of PDTFT/LECTS integration for multisensory fusion and high‐speed SNN learning.

Recent studies have shown that the first‐spike‐timing (FST), particularly within tactile neurons, plays a crucial role in encoding dynamic tactile information.^[^ ^12^, ^13^ ^]^ FST encoding offers higher information density and energy efficiency compared to rate encoding, significantly enhancing the processing capacity of SNNs for temporal information.^[^ ^14^, ^15^ ^]^ But the hardware implementation of FST encoding still remains challenging. Recently, FST encoding was validated using circuit strategies.^[^ ^11^, ^16^ ^]^ These researches demonstrate the advantages of FST encoding in reducing redundant data and enhancing real‐time processing for neuromorphic sensing. Nevertheless, the relatively high hardware complexity of circuit strategies could limit the broader application of FST encoding. Therefore, developing a device‐level approach provides a promising hardware strategy for implementing FST encoding in SNNs. Das et al. achieved optical spike timing encoding using dual‐TFTs based on MoS_2_, where temporal information was encoded by associating light intensity with the burst duration of neurons within a single device.^[^ ^3^ ^]^ Their experiments demonstrated the advantages of dual‐TFTs, such as charge accumulation and threshold triggering, indicating the potential for spatiotemporal encoding. However, FST encoding was not realized, mainly owing to the intrinsic physical limitations of TFTs, which lack the capability to emulate the neuron's resting state that determines the delay before the first spike fires (**Figure** **1**a).^[^ ^17^, ^18^, ^19^ ^]^ Specifically, considering the operation of an n‐type TFT, the gate voltage gradually increases to the specified value (U_0_) due to the parasitic capacitance. This resembles the gradual accumulation of membrane potential in neurons. Before the gate voltage reaches the subthreshold voltage (Sub_Vth) of the TFT, the transistor remains in a nearly off state (Figure 1b), corresponding to the resting period of neurons. As the gate voltage exceeds Sub_Vth, the drain current sharply increases, mimicking the first spike firing in neurons. Therefore, if Sub_Vth is lower than or exceeds U_0_, the device remains entirely silent or continuously fires, preventing the realization of FST encoding (Figure 1c). These suggest that an appropriate Sub_Vth provides the foundation for implementing FST encoding in TFT‐based systems. Moreover, to achieve spatial resolution and high integration of tactile signals, the deployment of sensor arrays is typically employed.^[^ ^20^, ^21^ ^]^ Nevertheless, device‐to‐device variations arising from manufacturing inconsistencies between units in an array pose a limitation in ensuring that Sub_Vth falls within the specific region, which is a necessary condition for successful FST encoding.

Precise FST encoding alone is insufficient for complex pattern recognition, requiring further processing by synaptic devices through modulation of pulse amplitude and timing to enable complex spatiotemporal learning in SNNs (Figure 1a). Hence, the design of high‐performance synaptic devices has been a central focus for achieving efficient tactile information processing.^[^ ^22^ ^]^ Among recent efforts, Kim et al. proposed a pressure‐electric co‐coupled memory transistor based on a TPU/[EMIM][TFSI] composite, where synergistic stimuli endowed tactile devices with tunable synaptic memory.^[^ ^23^ ^]^ Compared with single‐mode stimulation, this multimodal synergy enables higher feature separability at the same energy consumption, thereby enhancing the accuracy of information processing and decision‐making. Realizing such a unique feature requires a material that supports multi‐modal response. IGZO exhibits an low off‐state current and a high on/off ratio, along with excellent photo‐sensitivity, making it a promising material for multimodal coupled transistors.^[^ ^24^ ^]^ However, most reported IGZO devices focus on a single modality (either optical or electrical), and systematic studies on light‐electric coupling remain scarce. Exploring the coupling effect in IGZO could significantly enhance synaptic memory (Figure 1d). This enhancement arises from the introduction of light as an additional modulation pathway for conventional electrically driven synaptic weights, thereby shortening the training cycles of SNNs and reducing their energy consumption.

Here, we demonstrate a light‐accelerated SNN architecture integrating a pressure‐sensitive dual‐TFT (PDTFT) FST tactile encoder and light‐electric coupling transistor synapses (LECTS). The PDTFT was fabricated by monolithically integrating a PDMS/MWCNT pressure‐sensitive layer onto an IGZO dual‐TFT. FST tactile encoding was successfully achieved by adjusting the initially negative Sub_Vth to a positive one through bottom‐gate modulation, ensuring a resting period. Furthermore, the LECTS based on a GaO_x_/IGZO heterojunction transistor exhibits light–electric co‐activated synaptic plasticity resulting from a unique light‐electric coupling effect. Utilizing the FST tactile encoding capacity of the PDTFT and the light‐electric coupling effect of the LECTS, we developed a high‐accuracy status detection strategy for autonomous vehicles, achieving 98.4% classification accuracy for six statuses. Additionally, a smart navigation system for a walking robot was successfully validated with a navigation accuracy of 98.2% over 500 test paths via supervised learning with SNN. Moreover, the time consumption of SNN training was outstandingly reduced by 90.9%. This compact dual‐function system advances in‐sensor computing and oxide‐electronics‐based neuromorphic hardware for intelligent autonomous systems.

## 2 Results and Discussion

### 2.1 Sub_Vth Tuning in Dual‐TFTs via Bottom Gate

**Figure 2:**
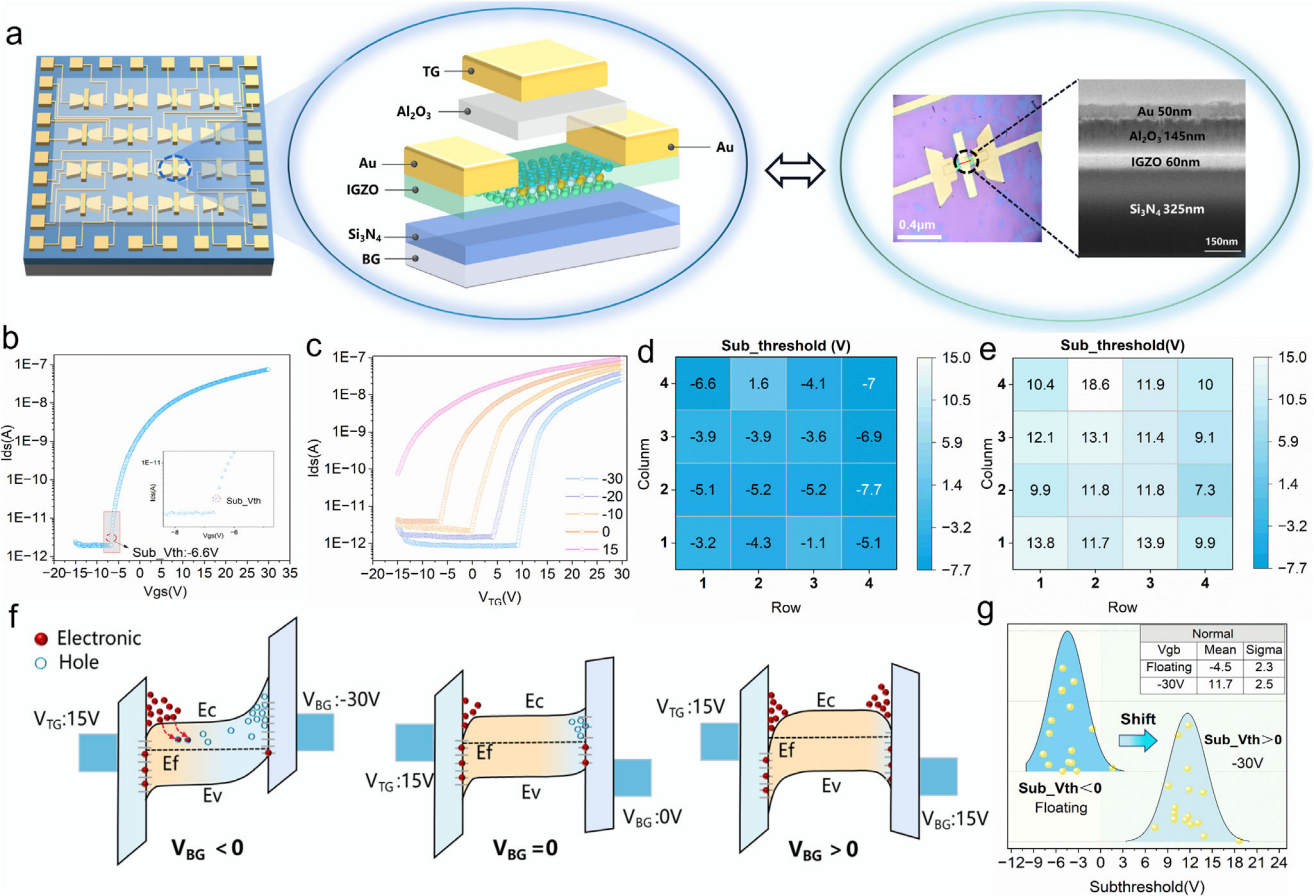
BG modulation of Sub_Vth in dual‐TFTs. a) Illustration of the device's multi‐layered structure, including structure schematic, OM, and cross‐sectional SEM images. b) The output transfer curve (ID‐VG) of dual‐TFTs, with the V_TG_ sweep ranging from −20 to 20 V and Vds fixed at 15 V. The red circle indicates the Sub_Vth position. c The ID‐VG curves under BG modulation at various voltages [−30, −20, −10, 0, and 15 V], measured with the same Vds ​ and V_TG_ ​ as in b). d) and e) represent the Sub_Vth distributions of a 4 × 4 device array without and with BG modulation, respectively. f) Mechanism of band energy variations affecting Sub_Vth at different V_BG_ values. g) Statistical analysis of the normal distribution of Sub_Vth for the 16 devices in the array, derived from the data presented in d) and e).

**Figure** **2**a illustrates the multi‐layered structure of the dual TFTs consisting of P‐type Si (Si^++^) / Si_3_N_4_ / IGZO / Al_2_O_3_ / Au, indicated through cross‐sectional scanning electron microscopy (SEM). The two gate electrodes, namely the top gate (TG) and bottom gate (BG), are used for modulating the IGZO channel. The dual TFT array consists of 16 devices arranged in a 4x4 configuration for encoding tactile information. Detailed fabrication procedures are available in the Methods section and Figure S2 (Supporting Information). The IGZO and GaOx films, confirmed by XPS and XRD analyses (Figure S26, Supporting Information), show the expected elemental compositions and amorphous structure.

The IGZO transistor behaves as an n‐type transistor when V_BG_ = 0. It operates in the cutoff and subthreshold regions when V_TG_ is below the threshold voltage (Vth). As V_TG_ increases, the drain current (Ids) remains near the off‐state in the cutoff region and then rises sharply as it approaches the subthreshold regime. At this point, the relative increment of Ids reaches its maximum, effectively mimicking the first spike firing of a neuron.^[^ ^25^ ^]^ The critical voltage is referred to as the subthreshold voltage (Sub_Vth), as shown in Figure 2b. Typically, the Sub_Vth of an n‐type TFT is negative, lying outside the top‐gate charging region (> 0 V). To ensure all devices enter the charging region, the BG is introduced along with TG to jointly modulate the IGZO channel. Figure 2c presents the *I*_D_‐*V*_G_ curves under various fixed BG voltages, demonstrating that a V_BG_ lower than −10 V is the key point to drive Sub_Vth into the charging region. The Sub_Vth for all TFTs within the array is measured in Figure 2d, revealing an average Sub_Vth of −4.45 V, which clearly lies outside the top‐gate charging region. The BG modulation mechanism can be explained using band theory. As shown in Figure 2g, when Vds is fixed and *V*_BG_ is floating, Ids predominantly depends on the top‐gate voltage (*V*_TG_). However, when a negative *V_BG_* is applied, electrons are repelled from the bottom‐gate side, causing the overall conduction band (Ec) of IGZO to shift upward. This upward band bending depletes the channel and increases the energy barrier for electron transition, thereby reducing the electron density and limiting the channel conductivity. Consequently, the Id‐Vg shifts to the right, indicating a positive shift in the threshold voltage (Vth increases); the opposite trend occurs for a positive *V_BG_*. To quantify this shift across the device array, we measured the Sub_Vth distribution with a BG bias of −30 V (Figure 2e). As summarized in Figure 2g, the average Sub_Vth shifts from −4.5 to 11.7 V, with a nearly unchanged standard deviation (σ) remaining constant at 2.3–2.5 V. These results highlight that BG modulation can precisely and effectively drive Sub_Vth into the charging region without deteriorating uniformity, thereby providing the necessary condition for simulating the resting period.

### 2.2 Single‐Device Implementation of FST Tactile Encoding

**Figure 3:**
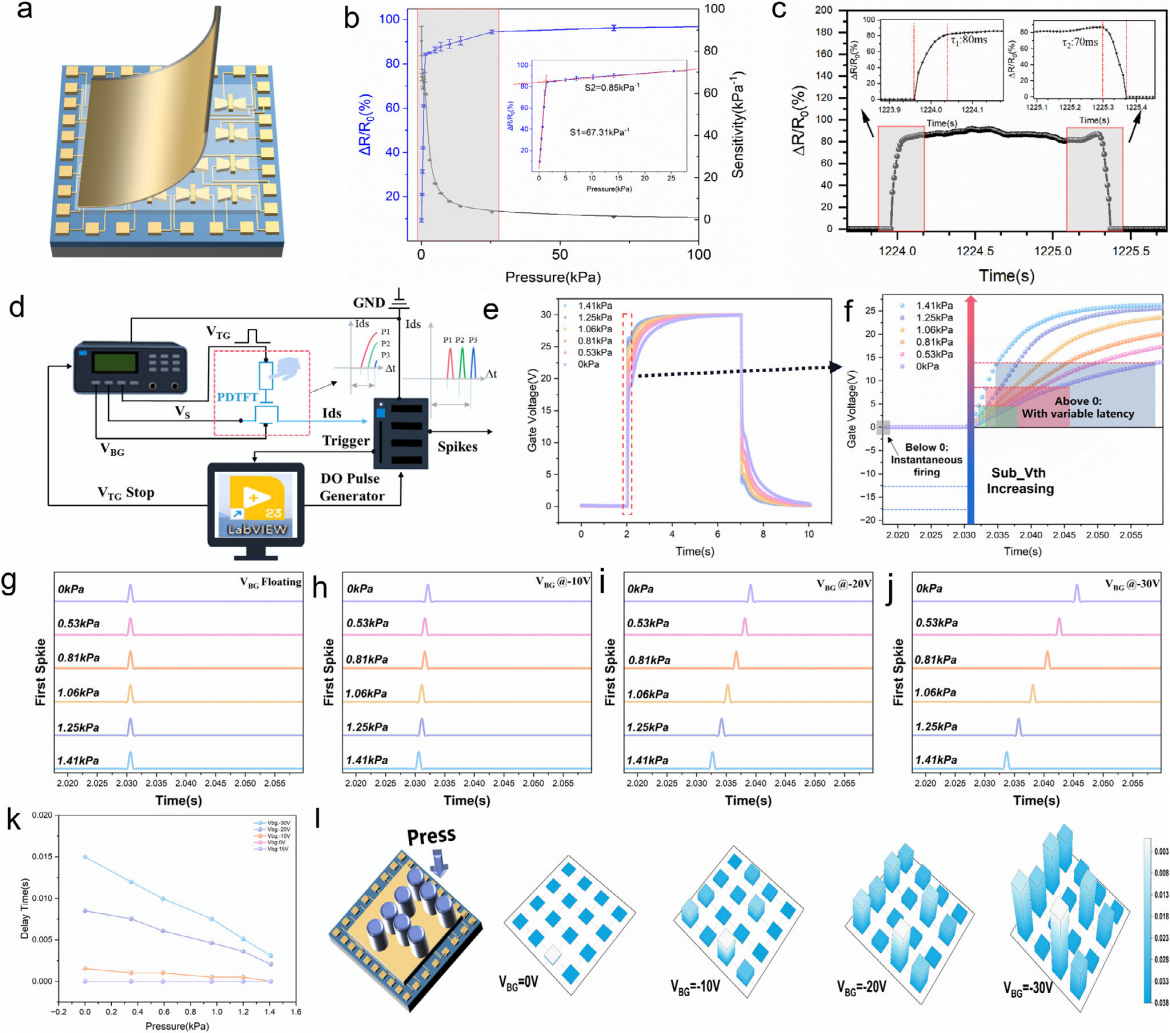
The piezoresistive characteristics of PDs and their integration with dual‐TFTs for tactile FST encoding. a) Schematic diagram of PDTFT. b) The PDs’ change in resistance rate (∆R/R0) with pressure, where the sensitivity (S) can be calculated from the slope of the stage. c) 600‐cycle pressure testing for PDs using a stepper motor at a frequency of 0.33 Hz. d) Schematic illustration of the FST testing principle of the PDTFT. e) The V‐T charging and discharging curves. A voltage pulse [30 V, 5 s] was applied to the TG of PDTFT at various pressures. The voltage was measured by connecting the anode of the device to the PD's top electrode and the cathode to the source of the transistor, forming an RC circuit for voltage charging and discharging measurements. f) The influence of the Sub_Vth region on the pressure‐dependent delay time differences of first spike generation. g–j) The FST results of PDTFT under BG modulation at 0, −10, −20, and −30 V, respectively. k) The relationship between the first spike delay time and applied pressure under different BG modulation conditions. l) "F"‐shaped cylindrical probe applied mechanically to the surface of the PDTFT within a 4 × 4 array. With V_BG_ set to −30 V and Vds at 15 V.

To enable tactile sensing, a pressure‐sensitive layer composed of PDMS/MWCNTs (PDs) was integrated onto the top gate of the dual‐TFT structure (**Figure** **3**a). The resulting integrated device, a pressure‐sensitive dual‐gate thin‐film transistor, is referred to as PDTFT. The PDs were first independently characterized to evaluate their pressure‐sensing performance. The cross‐sectional SEM images of the PDs (Figure S3, Supporting Information) reveal a uniform textured PDMS with uniformly embedded MWCNTs. The piezoresistive mechanism (Figure S4, Supporting Information) involves pressure‐induced changes of the MWCNT network: at zero pressure, randomly distributed MWCNTs form limited conductive pathways in PDMS, resulting in high initial resistance. Low pressure causes slight PDMS deformation, rapidly increasing contact points, which sharply enhances conductivity and yields high sensitivity. As pressure increases further, the network densifies and approaches saturation, leading to diminished resistance variation and decreased sensitivity.^[^ ^26^ ^]^

To assess the sensitivity (S) of the PDs, we measured the relative resistance change (Δ*R*/*R*_0_) under varying pressures (Δ*P*). The detailed calculation for S is provided in Note 1. The PDs exhibited pressure‐dependent sensitivity, with S = 67.31 kPa^−1^ below 1.54 kPa and 0.85 kPa^−1^ at higher pressures (Figure 3b). Response/recovery times were 80/70 ms, demonstrating rapid tactile signal conversion (Figure S5, Supporting Information) and stable performance in a 600‐cycle robustness test (Figure 3c).

Next, we investigated the FST encoding performance of the PDTFT, which integrates a dual‐TFT with PDs. Figure 3d illustrates the encoding test system for the PDTFT. A gate voltage pulse is applied to the PDTFT by a signal generator, while the NI acquisition system continuously monitors Ids. Upon detecting the first spike, a digital output pulse was triggered to stop the gate bias, and the extracted first‐spike latency (Δt) is used as the temporal code of the input stimulus intensity, which is then output through the DO port of the acquisition card. Then, Figure 3e shows the charging/discharging *V‐T* curves under a V_TG_ pulse of [30 V, 5 s], measured at different pressure levels. This behavior can be described by the following equation^[^ ^27^ ^]^:

(1) $$\begin{array}{c} V\left(t\right)=V_{target}\left(1-e^{-\frac{t}{RC}}\right) \end{array}$$

where the PDs act as a resistive film, which forms an RC circuit with the top gate (TG) of the TFT. Due to the intrinsic RC delay, the target TG voltage (*V_target_*) corresponds to the device's Sub_Vth and rises gradually over time rather than instantaneously. This suggests that pressure‐induced resistance changes (ΔR/R_0_) directly affect the time required to reach the Sub_Vth (*V_target_*) of the PDTFT. Specifically, higher pressure accelerates the charging process, reducing the time to reach Sub_Vth and resulting in an earlier first spike. However, previous studies show that most IGZO TFTs possess a negative Sub_Vth, enabling detectable Ids even at zero TG voltage.^[^ ^28^, ^29^ ^]^ This leads to continuous firing without a resting period, making it difficult to distinguish pressure levels based on first‐spike delay (Figure 3d). By shifting the Sub_Vth to a positive range via different negative BG modulation (V_BG_<0 V), a clear resting period appears, allowing effective temporal encoding (Figure 3h–j). As V_BG_ decreases from −10 to −30 V, the resting phase becomes progressively longer, during which the pressure‐dependent resistance of PDs directly maps pressure differences to spike‐timing delays. At V_BG_ = −30 V, the resting period extended from ≈2 to 15 ms, yielding a Δt of 11.8 ms between 1.41 and 0 kPa with distinct, non‐overlapping spike peaks, thus achieving the highest temporal resolution. Furthermore, the complete first‐spike encoding process under various BG modulations is presented in Figure S7 (Supporting Information). Then, the relationship between pressure and first spike delay timing (∆t) under various V_BG_ modulations is shown in Figure 3k. As pressure increases, ∆t becomes shorter, which is consistent with the biological observation that stronger stimuli reduce action potential latency in neurons.^[^ ^14^ ^]^ Notably, FST encoding performance is strongly dependent on the specific value of V_BG_. When V_BG_ is set to 15 or 0 V, ∆t remains unchanged across varying pressures, indicating that insufficient modulation fails to produce effective spike timing responses. In contrast, at −20 and −30 V, Δt exhibits a pronounced monotonic increase with decreasing pressure, showing highly linear behavior with a markedly steeper slope. Furthermore, the physical model of the PDTFT operation is detailed in Note S2 (Supporting Information). The relationship between Δt and P indicates that the resistance of the PDs (*R*_0_) is in the low‐resistance region, far lower than the parasitic resistance of the IGZO TFT (*R*_c_), i.e., *R*_0_≪*R_c_*. Finally, a 4 × 4 array validated the pressure‐based FST encoding. A needle probe applied a pressure of 2.45 kPa (Figure 3i), and as V_BG_ decreased, the first spike timing differentiation across the array became more distinct, confirming that BG modulation enables spike timing encoding across all devices in the array, even when the initial Sub_Vth does not lie in the expected region, demonstrating the universality of the strategy in FST encoding. Moreover, considering the continuous PD film, array crosstalk was simulated using COMSOL multi‐physics mechanical‐electrical coupling modules, as shown in Figures S9–S12 (Supporting Information). As detailed in Note S3 (Supporting Information), the crosstalk decays rapidly with distance. For the device pitch of 1.5 mm, the crosstalk remains as low as 0.61% even under a high pressure of 100 kPa (actual 1.4 kPa), far below the typical engineering tolerance of 1–2%, indicating that the weak coupling between array pixels is insufficient to induce noticeable electrical crosstalk.

### 2.3 Light–Electric Co‐Activated Synapse with Light‐Electric Coupling Effect

Sensory neurons convert external physical stimuli into spike trains, with the first spike timing reflecting distinct temporal characteristics, and subsequently transmit the signals to downstream neurons via synaptic connections.^[^ ^30^ ^]^ The postsynaptic current amplitude is shaped by two key factors: spike amplitude and timing. Stronger synapses produce larger currents per spike, accelerating membrane potential buildup. Meanwhile, closely timed spikes summate more effectively than dispersed ones. Together, these factors enable neurons to perform spatiotemporal integration of input patterns.^[^ ^19^ ^]^ Specifically, variations in synaptic strength and spike timing allow neurons to distinguish between distinct sequences of activity across both time (e.g., rapid vs delayed inputs) and space (e.g., inputs from different pathways).^[^ ^31^ ^]^ This dual modulation mechanism is fundamental for the brain's capacity to recognize and learn complex temporal patterns.

**Figure 4:**
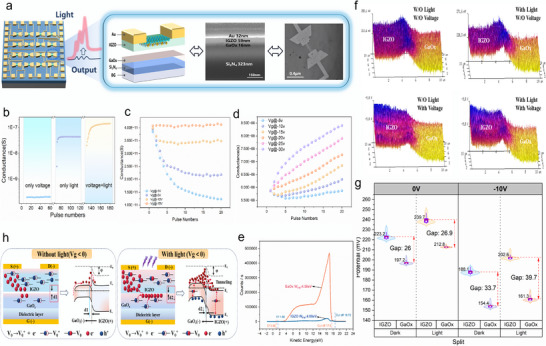
The performance and characteristics of the light‐electric coupling effect in LECTS. a) Illustration of the multi‐layered structure of the LECTS, including the device schematic, cross‐sectional SEM, and optical images. b) Three stimulation modes are applied to the BG of LECTS to investigate its electrical response: light‐only, electrical‐only, and light‐electric coupling under different numbers of applied voltage pulses [−15 V, 0.2 s] on the BG, with a read voltage of [−0.2 V, 0.1 s]. c) Conductance evolution under multiple electrical pulses at different gate voltages under dark conditions. d) Conductance evolution under pulsed stimulation at different gate voltages under light conditions, with a read voltage of −0.2 V applied for 0.1 s. e) The work functions of GaO_X_ and IGZO were determined using ultraviolet photoelectron spectroscopy (UPS), with the calculation method.^[^ ^34^ ^]^ f) KPFM surface potential distributions of IGZO/GaOx under different excitation modes: dark/no gate bias, light only, gate bias only, and light + gate bias. g) Statistical distribution of the IGZO/GaOx surface potential derived from Figure 4f. h) Mechanism of the light‐electric coupling effect, analyzed via the energy band structure of the IGZO/GaO_X_ heterojunction under light and dark conditions.

Given the crucial role of synaptic weight modulation, this section introduces the high‐performance light‐electric co‐activated synaptic characteristics of the LECTS for FST processing. **Figure** **4**a shows the structure of the LECTS device based on a light‐electric coupling effect, which differs from traditional artificial synapses that respond solely to light or electrical stimulation. The LECTS device structure of P‐Si^++^/Si_3_N_4_/GaO_x_/IGZO/Au is similar to the above‐discussed dual‐TFT configuration. A GaO_x_ layer is inserted below the IGZO to construct a GaO_x_/IGZO heterojunction, providing dual functionality as a barrier for electron transport and a photogenerated electron source. In Figure 4b, the device exhibited different response modes under three simulations: light‐only, electric‐only, and light‐electric synergetic stimulations. When only negative electric pulses were applied to the BG of the LECTS, Ids remained unchanged, indicating the absence of memory characteristics. Under light‐only stimulation, a weak and short‐lived memory was observed due to rapid saturation. Notably, light‐electric synergetic stimulation leads to the highest EPSC and pronounced memory behavior, far surpassing the responses observed under single‐mode stimulation. The outstanding memory windows in the *I*_D_‐*V*_G_ curves shown in Figure S14 (Supporting Information) further showcase the light‐electric co‐activated synaptic feature of the LECTS, providing essential characteristics for the applications in multi‐sensory fusion and supervised learning.

In order to probe the light‐electric coupling mechanism in the LECTS, the channel conductance of the LECTS device stimulated by a train of electrical pulses on the gate was recorded at different gate voltages under dark/illumination conditions. Figure 4c shows the conductance change under dark conditions, where the conductance gradually decreases with increasing pulse number. This indicates that the density of free electrons in IGZO is progressively decreased owing to the defect traps in IGZO. However, as the gate voltage amplitude increases (such as −15 V), the decrease of conductance becomes less pronounced, suggesting that a stronger electric field promotes the replenishment of electrons from GaOx to IGZO. Figure 4d illustrates the conductance change under illumination. At low voltages (−5 to −15 V), the conductance initially decreases and then increases with the number of pulses, indicating that electron accumulation must reach a critical threshold before injection from GaOx into IGZO occurs. With increasing the voltage or light intensity (Figure S17, Supporting Information), this threshold gradually decreases and eventually disappears.

To further investigate the mechanism, the work function difference between GaO_x_ (4.58 eV) and IGZO (4.08 eV) confirms the formation of a GaO_x_/IGZO heterojunction (Figure 4e). This energy offset leads to an upward band bending under an external electric field, resulting in a barrier (ϕ_*B*_) that suppresses the electron migration from GaO_x_ to IGZO. The KPFM results (Figure 4f,g) reveal the distinct surface potential changes of the IGZO/GaOx heterojunction under different modes (measurement method described in Note S5, Supporting Information). In the initial state, IGZO (223.2 mV) shows a higher potential than GaOx (197.2 mV), indicating a lower work function of GaOx, which is consistent with the data shown in Figure 4e. Under light‐only conditions, both the potentials of IGZO (239.7 mV) and GaOx (212.8 mV) rise as shown in Figure 4g. Under the electric‐only bias with the external field aligning with the built‐in field, the interfacial potential difference, thus barrier height (ϕ_*B*_​), are obviously increased to 33.7 from 26.0 mV. With light–electric synergetic stimulation, the potential difference between IGZO and GaOx reaches the maximum (39.7 mV). This hinders the electron transfer from GaOx to IGZO due to enhanced band bending (Figure 4h). A large number of electrons, driven by the negative bias, gradually accumulate at the GaOx/IGZO interface but are confined by the barrier ϕ_*B*_ (Figure 4h). According to Equations (2) and (3), the barrier width Δ*x* decreases, implying an increased probability of quantum tunneling.^[^ ^32^ ^]^

(2) $$\Delta x=\sqrt{\frac{2\varepsilon_{0}\varepsilon_{r}\left(V_{bi}-V_{a}\right)}{qN_{D}}}\;$$

(3) $$P=\exp\left[-\frac{8\pi }{3}\left(\frac{2_{m}^{*}}{h^{2}}\right)1/2E_{2}^{1/2}\Delta x\right]$$

where *V_a_* represents negative bias voltage, and N_D_ is the electron concentration. *P* denotes the tunneling probability, $m_{n}^{*}$ is electron effective mass, E_*g*_ is bandgap, and Δ*x* is the effective barrier thickness, which decreases with electron accumulation in GaOx.^[^ ^32^ ^]^ Therefore, the light‐electronic coupling mechanism can be illustrated in Figure 4e. Under dark conditions, a negative gate voltage drives electrons from the Si_3_N_4_ and GaO_x_ layers toward the IGZO channel. However, due to GaO_x_s high electron affinity, it does not readily release free electrons.^[^ ^33^ ^]^ As a result, a relatively high gate voltage (e.g., Figure 4c Vg@‐30 V) is required to achieve a dynamic balance between electron injection and trapping in IGZO. In this regime, the conductance becomes insensitive to gate voltage, indicating a lack of synaptic feature. Upon illumination, a large number of photo‐generated electrons are produced in the GaOx and IGZO layers. As the electron concentration at the GaOx/IGZO interface increases, the tunneling probability increases due to the decrease in the effective barrier thickness as stated above. Thus, the field‐induced tunneling transport happens for the IGZO/GaOx heterojunction. This enables substantial electron injection into the IGZO channel. These injected electrons increase the channel conductance, while electrons captured by oxygen vacancies in GaOx result in persistent memory retention behavior.

**Figure 5:**
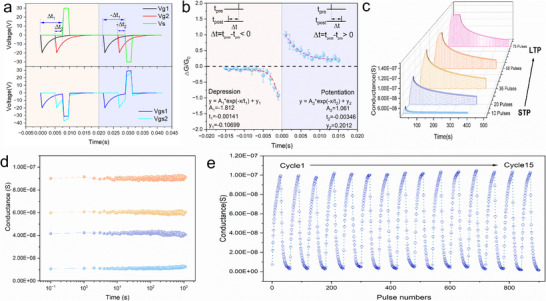
The synaptic weight plasticity performance of LECTS. a) Overlapping strategy of Vs and Vg waveforms for STDP realization. ΔT represents the relative spike time between Vs and Vg. When Vs > 0 and Vgs < 0, LTP occurs; when Vs < 0 and Vgs > 0, LTD of synapses is realized. b) The weight change rule based on the STDP for LECTS. c) The change of conductivity over time under different numbers of applied voltage pulses [−30 V, 0.5 s] on the BG, with a read voltage of [−0.2 V, 0.1 s]. d) Retention characteristics were measured under four distinct conductance states. e) Operational fatigue test of LECTS over 15 cycles under light conditions. Each cycle includes 35 negative pulses [−30 V, 0.5 s] for LTP with a read pulse [‐0.2 V, 0.1 s], and 35 positive pulses [30 V, 0.5 s] for LTD with a read pulse [0.2 V, 0.1 s].

Spike‐timing‐dependent plasticity (STDP), a fundamental learning rule for updating synaptic weights based on spike timing, plays a critical role in mapping the time difference between spikes.^[^ ^35^, ^36^ ^]^ Thus, mimicking the STDP rule is essential for effectively encoding temporal information. The BG of the device is used as the pre‐neuron pulse input with spike time. *t*_pre_, and the source is used as the post‐neuron pulse input with spike time *t*_post_, with the time difference between the two spikes denoted as ∆T, **Figure** **5**a illustrates the specific details of the signal input. The simulated STDP behavior is described by the following equation:^[^ ^37^ ^]^

(4) $$\Delta w=\left\{\begin{array}{c} \mathrm{Vs}*e\frac{-\left(t_{\mathrm{pre}}-t_{\mathrm{post}}\right)}{\tau },\Delta T=\left|t_{\mathrm{pre}}-t_{\mathrm{post}}\right|\;\mathrm{Vs}>0\left(\mathrm{LTP}\right)\\ \mathrm{Vs}*e\frac{-\left(t_{\mathrm{pre}}-t_{\mathrm{post}}\right)}{\tau },\Delta T=\left|t_{\mathrm{pre}}-t_{\mathrm{post}}\right|\;\mathrm{Vs}\langle >\rangle 0\left(\mathrm{LTD}\right) \end{array}\right.$$

In contrast to biological STDP, where ΔT represents the exact temporal relationship between pre‐ and post‐synaptic spikes, the conductance of our artificial synapse is governed by voltage polarity, not by genuine spike‐timing differences. Specifically, a positive source voltage (Vs​>0, equivalent to ΔT>0) is applied to simulate long‐term potentiation (LTP), while a negative source voltage (Vs​<0, equivalent to ΔT<0) is used to induce long‐term depression (LTD). The results in Figure 5b indicate that light–electric co‐activated synapse stimulation effectively emulates the STDP behavior, whereas single‐mode stimulation fails to reproduce this essential plasticity. Figure 5c shows the memory performance of the device with different numbers of pulses. As the pulse counts increase, the device transitions from short‐term potentiation (STP) to long‐term potentiation (LTP). Moreover, Figure 5d shows that the device exhibits good long‐term retention, with four distinct conductance states maintained for over 1000 s. Finally, to verify long‐term reliability, the device underwent 15 LTP/LTD training cycles (Figure 5e), demonstrating excellent synaptic stability and anti‐fatigue performance. These results confirm that the LTCTS enables robust, repeatable long‐term memory, supporting reliable synaptic weight updates for neuromorphic applications.

### 2.4 Distance‐Dependent State Detection through PDTFT/LECTS with Multi‐Sensory Fusion

In autonomous driving, the vehicle's driving state can be assessed based on the relative distance to the preceding vehicle, a method known as Distance‐Dependent State Detection. This approach enhances situational awareness, allowing the system to identify acceleration, deceleration, and stopping behaviors of the lead vehicle.^[^ ^38^ ^]^ As a result, it helps predict potential risks and improves decision‐making, contributing to safer driving conditions.^[^ ^39^ ^]^

**Figure 6:**
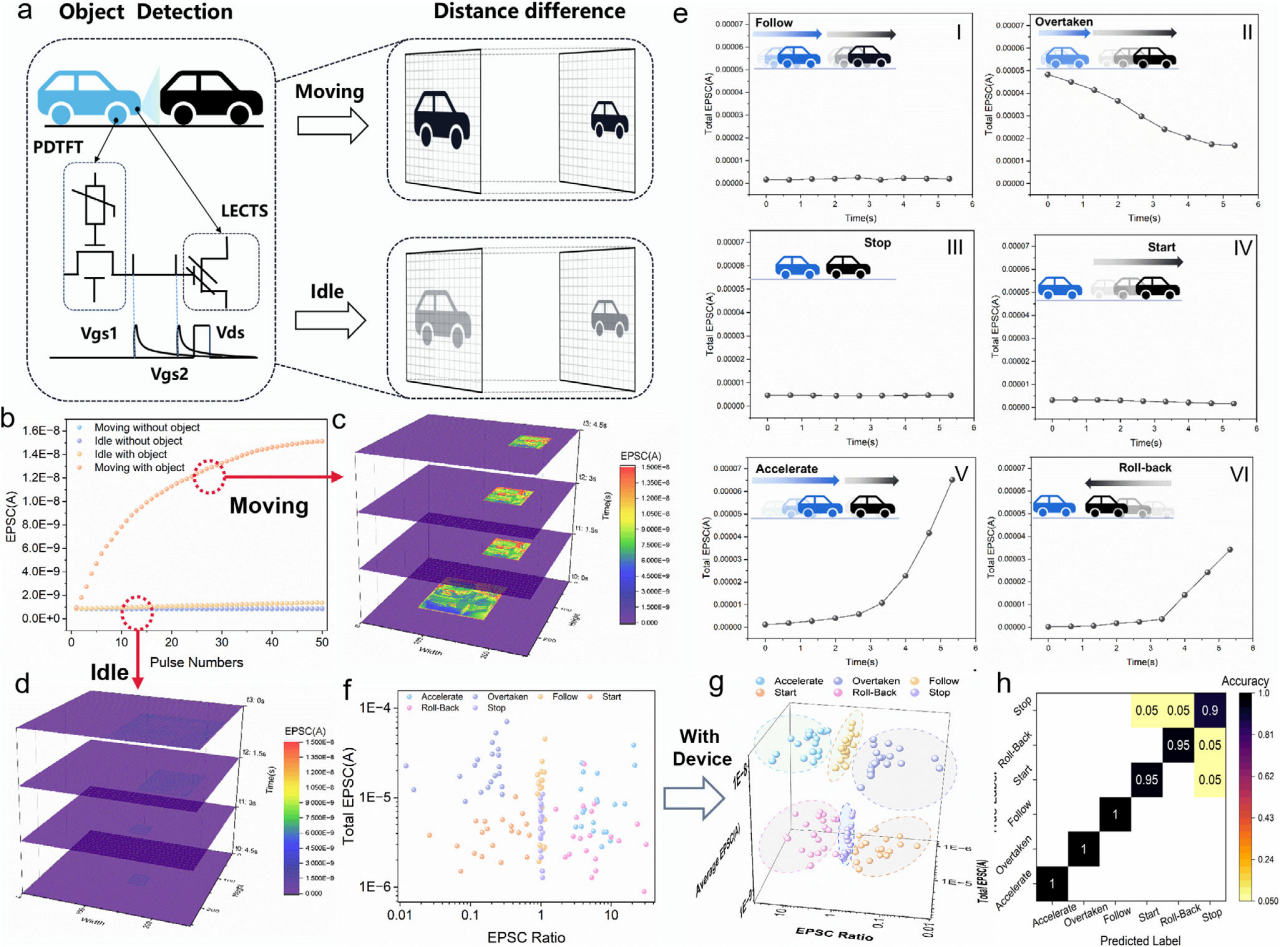
Application in distance‐dependent state detection. a) Schematic diagram of the PDTFT/LECTS hardware system for relative distance to the preceding vehicle and the current state of the vehicle. b) Spikes generated by the PDTFT under pressure and no‐pressure conditions are transmitted to the LECTS under light and dark states, resulting in four distinct EPSC responses. c,d) show the vehicle status as "Moving" and "Idle," respectively, determined from the relative distance of the preceding vehicle using a 125 × 125 LECTS array, as introduced in b). In c), the preceding vehicle moves from near to far, leading to a decrease in image size (total EPSC), where the higher EPSC corresponds to an "Overtaken" state. In contrast, d) illustrates a "Roll‐back" state, where the preceding vehicle approaches from far to near, resulting in lower EPSC. e) Processing flow for determining six vehicle driving states. The video is divided into 9 frames, and the total EPSC for each frame is calculated from the 125 × 125 LECTS array. f,g) show the classification results of six vehicle states across 120 samples, without and with the proposed device, respectively. In f), only two features—EPSC Ratio and Total EPSC—are used, whereas g) includes an additional feature: Average EPSC. h) Confusion matrix for distance‐dependent state classification using Support Vector Machine (VSM) on 120 samples.

PDTFT/LECTS, as an integrated device combining pressure encoding and light‐electric coupling features, is highly suitable for monitoring changes in the lead vehicle's distance and the current vehicle's movement, with the synchronization of these two features aiding decision‐making for the driving state. To simulate Distance‐Dependent State Detection, we developed a hardware system, as shown in **Figure** **6**a. This system consists of a single PDTFT mounted on the tire and a 125 × 125 array of LECTS units positioned at the front of the vehicle. This system consists of a single PDTFT mounted on the tire and a 125 × 125 array of LECTS units positioned at the front of the vehicle. The PDTFT converts the pressure signal into an electrical response, which is processed by the NI Port and output as discrete voltage pulses directly applied to the gate of the LECTS, while the source electrode (Vs) is held at a fixed reference potential. The LECTS then converts the timing of these gate voltage spikes, which reflect the magnitude of the applied pressure, into pressure‐dependent source currents (Ids), thereby enabling tactile sensing to detect vehicle movement. In this configuration, the PDTFT functions as a pressure encoder, categorizing the vehicle's state as either “Idle” or “Moving,” while the LECTS array integrates this information to perceive the presence and relative distance of the lead vehicle. In this configuration, the PDTFT functions as a pressure encoder, categorizing the vehicle's state as either “Idle” or “Moving”, while the LECTS array integrates this information to perceive the presence and relative distance of the lead vehicle. Due to the pressure encoding capabilities of the PDTFT, a “Moving” state triggers earlier spikes, which, after synaptic processing by the LECTS, result in a higher EPSC, whereas the “Idle” state yields much lower EPSC values. Moreover, due to the light‐electric coupling effect of LECTS, the units generate larger EPSC values when exposed to light (with object) compared to when no light is applied (without object). Figure 6b shows the changes in EPSC for a 1 PDTFT/1 LECTS under four combinations of light (on/off) and pressure (on/off) conditions. The results demonstrate that “Moving with light” reaches an EPSC of 1.5 × 10^−8^ A, “Idle with light” reaches 1.73 × 10^−9^ A, and “Idle/Moving without light” shows EPSC values lower than 10^−9^ A. These findings indicate that distinct driving scenarios, including the combined states of current and preceding vehicles, can be clearly differentiated based on their corresponding EPSC values.

To further differentiate additional states, the LECTS array is capable of sensing the preceding vehicle's image and assessing the relative distances between the two vehicles by monitoring the total EPSC variation. This enables the detection of six states, such as “Accelerate” (Figure 6c) and “Roll‐Back” (depicted in Figure 6d). As observed, in the “Accelerate” state, the total EPSC (image size) decreases over time due to the increasing distance. Meanwhile, in the “Roll‐Back” state, since the current vehicle is in the Idle state, the total EPSC is lower than that of the “Accelerate” state and increases over time as the distance decreases. The raw captured images of these two states are shown in Figure S20 (Supporting Information). The six states were derived from the collected videos, which were divided into 9 frames. The total EPSC for each frame was then calculated using the method outlined in the Methods section. The results are shown in Figure 6e (I–VI). The states can be summarized into three categories based on the distance change: 1) Distance increasing (Figure 6e (II, IV)), where the total EPSC exhibits a negative correlation with the frame number; 2) Distance decreasing (Figure 6e (V, VI)), where the total EPSC shows a positive correlation; and 3) Constant distance (Figure 6e (I, III)), where there is little to no change in EPSC. Finally, we used more video samples (20 samples per state, total = 120) to test the recognition accuracy. The results, shown in Figure 6g,h, clearly demonstrate that the PDTFT/LECTS system enables the distinction of the six states due to its pressure and light fusion feature. In contrast, without this hardware system, the six states cannot be accurately differentiated, as shown in Figure 6f. This enhancement introduces an additional dimension to the original 2D feature space, significantly improving state differentiation with an accuracy of 98.4%. These results demonstrate the immense potential of the PDTFT/LECTS device to facilitate real‐time state detection in autonomous driving systems, thereby enhancing safety and navigation efficiency through robust intelligent decision‐making.

### 2.5 Light‐Enhanced Supervised Learning for Global Path Navigation via SNNs

**Figure 7:**
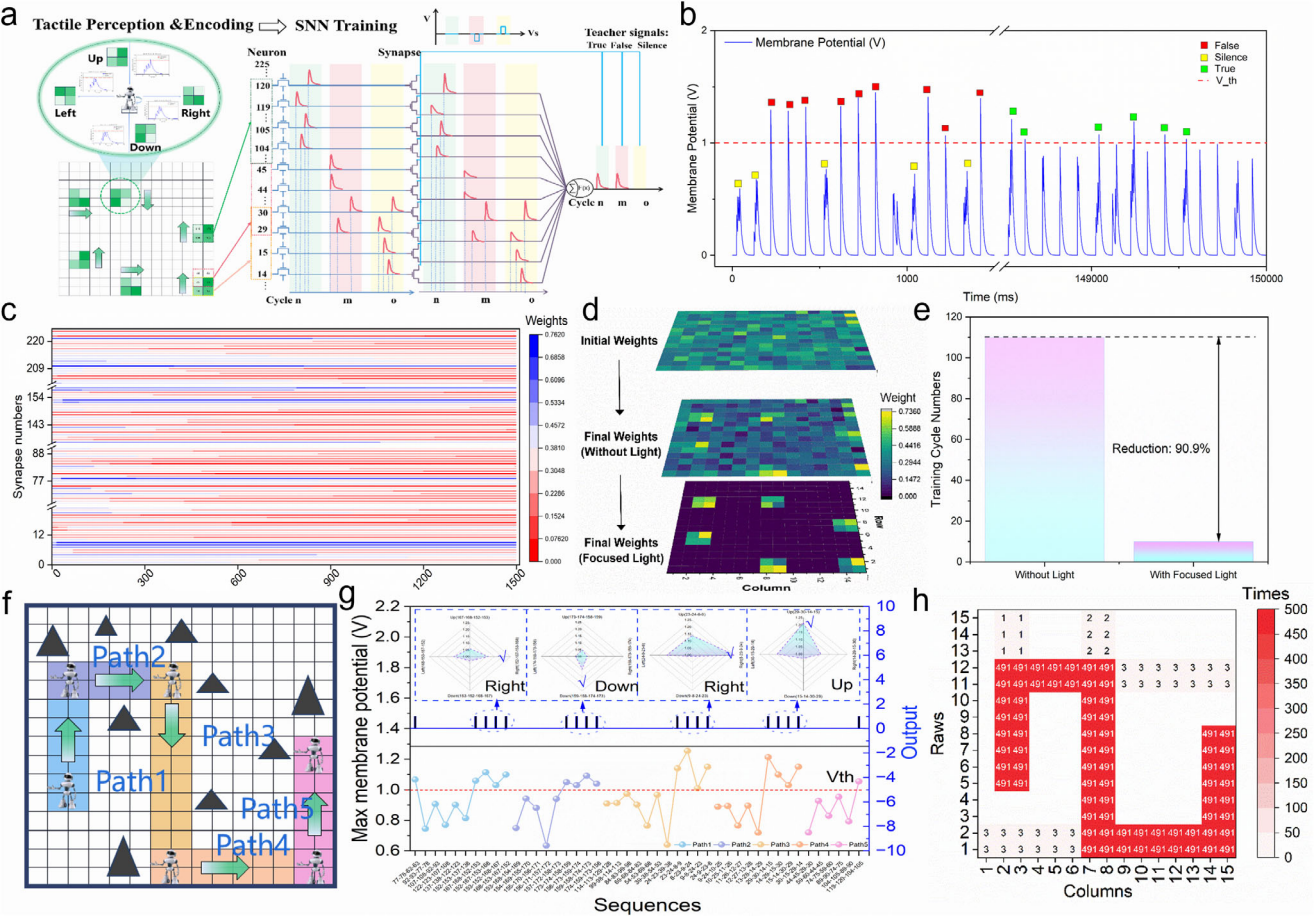
Application in global path navigation based on spatiotemporal detection. a) Schematic diagram of the SNN architecture for pressure sensing, FST encoding, and spatiotemporal learning using PDTFT/LECTS. (b) The membrane potential variation of the postsynaptic neuron over 150 000 ms (100 ms/frame) and the teacher signal determination. c) The weight update process of the 225 LECTS during SNN training over 1500 frames. d) Weight distribution of the 225 LECTS devices before and after training with different strategies. e) Training time of the SNN with and without focused light illumination. f) the designed pathway for the walking robot. g) the entire process of the robot moving along the designed path: going straight, stopping, decision‐making, and turning. h) the accuracy of the robot moving along the designed pathway.

In this section, we aim to highlight the capability of LECTS in spatiotemporal learning. A simulation model was constructed with a 15 × 15 PDTFT/15 × 15 LECTS/1 Neuron, as shown in **Figure** **7**a. To model the robot's interaction with its environment, the robot's feet are positioned within a 2 × 2 block, with each unit consisting of a PDTFT/LETCS device. The block exhibits distinct pressure distribution detection and encoding, which vary based on its orientation and movement position. These blocks can be regarded as different sequences of spike timing, encoding the pressure distribution after PDTFT processing. Building on this model, the next step in the process is SNN training, where a random block, containing four spikes with different timings, is input at each frame. This includes six target blocks used for reinforcement learning, which correspond to turning nodes along a predefined path. The teacher supervision scheme is employed during the learning process, providing feedback based on the integration and threshold comparison of EPSC through post‐synaptic neuron LIF behavior, which leads to weight updates in the LECTS. The learning rules are as follows: 1) If the target sequence does not fire, the feedback is labeled “silence,” and the weight undergoes LTP. 2) If the target sequence fires, the feedback is labeled “true,” and the weight remains unchanged. 3) If other sequences fire, the feedback is labeled “false,” and the weight undergoes LTD. These rules trigger the synaptic update condition after each frame is evaluated.

The SNN training process is commonly used as an indicator to evaluate the performance of the model's training. Figure 7b shows the output of the postsynaptic neuron EPSC over 1, 500 frames. As observed, the previous 10 frames primarily exhibit ′false′ feedback, indicating that the initial random 2 × 2 block triggers erroneous postsynaptic neuron spikes. This reflects that, during the robot's movement along the planned path, it triggers a false signal due to non‐turning nodes. These errors must be eliminated to retain only the nodes that need to be turned. After training, the last 10 frames successfully achieve this goal. Meanwhile, Figure 7c shows the weight update process for the 225 LECTS units, which follows the STDP rule depicted in Figure 5b. The updated weight array's visual results can be seen in Figure 7d. The training process successfully maps the target sequence into the weights. The final distribution of the trained weights aligns with the pressure distribution of the robot's feet. This indicates that the synaptic weights after training exhibit a positive correlation with spike timing, facilitating effective spatiotemporal recognition.^[^ ^40^ ^]^ However, as shown in Figure S23 (Supporting Information), most of the learning time (110 frames) is spent on LTD of non‐turning nodes, with only a small portion used to enhance the target sequence. This inefficiency incurs excessive time and energy expenditure during training, undermining the overall efficiency of the learning process.

To address these inefficiencies, LECTS leverages a unique light‐electric coupling feature, which accelerates spatiotemporal learning. Specifically, a focused light strategy is first employed to rapidly increase the weights of target nodes, followed by weight modulation through post‐encoding pulses. Selective illumination of turning nodes rapidly suppresses non‐turning nodes via conductance reduction in unlit regions. This strategy enhances the synaptic signal‐to‐noise ratio, effectively suppressing non‐target responses and reducing erroneous triggers along the robot's path (Figure 7d). Moreover, as shown in Figure 7e, the proposed approach completes reinforcement training within 10 cycles compared to 110 cycles with conventional synapses, achieving a 90.9% reduction in learning time (from 110 to 10 s). The definition of training time and convergence criteria in SNNs is provided in Note 7. Therefore, the significant reduction in learning time and the enhanced robustness of the final weight map are crucial for improving the efficiency of spatiotemporal learning. These advancements demonstrate the potential of LECTS to lower energy consumption, making it ideal for large‐scale adaptive decision‐making in complex dynamic environments.

To further demonstrate the potential applications of spike timing encoding and efficient learning in SNNs, we explore its application to global path navigation as shown in Figure 7f. The movement trajectory depicted in Figure 7g, the robot's movement within a 15 × 15 array, continuously traveling in the predefined direction until neuron spikes are generated. The robot only alters its path when it reaches a turning node (target sequence), at which point the postsynaptic neuron triggers a spike, causing the robot to stop moving and make directional decisions. Since the four directions represent four different combinations of sequences, for example, the sequence for up is 29‐30‐14‐15, for right is 14‐29‐15‐30, for down is 15‐14‐30‐19, and for left is 30‐15‐29‐14. All four sequences can trigger the neuron's threshold, but only the target sequence (designed directions) generates the highest EPSC (Figure S25, Supporting Information for the EPSC of each direction). Therefore, the next direction of travel is determined autonomously, and the process continues until the predefined path is completed. To validate the robot's performance in traversing the planned path multiple times, a path traversal test was conducted for 500 cycles, as shown in Figure 7h, achieving an accuracy of 98.2% in following the planned path. These results underscore the potential of the LECTS‐based system in real‐time neuromorphic path navigation. By leveraging spatiotemporal learning and adaptive weight modulation, the system accelerates path convergence and enhances robustness, showcasing the feasibility of hardware‐implemented neuromorphic intelligence for autonomous navigation in dynamic environments.

## 3 Conclusion

In summary, this study demonstrates a neuromorphic architecture that overcomes two challenges in SNNs: precise spatiotemporal sensory encoding and high‐efficient synaptic processing. The pressure‐sensitive IGZO‐based dual‐TFT encoder achieves millisecond‐level FST tactile encoding using a single device, and directly converts pressure stimuli into spike trains with a temporal resolution of 0.1–5 ms. The key advance lies in the effective device‐level hardware implementation strategy for FST spike encoding by the dual‐gate control, which dynamically modulates the Sub_Vth within a specific range to emulate biological neuronal resting states. Subsequently, a light–electric co‐activated synaptic plasticity is validated using a GaO_x_/IGZO heterojunction transistor. The photosensitive heterojunction channel in LECTS results in a unique light‐electric coupling effect. Unlike conventional synapses with single‐mode responses induced by light or electric stimuli alone, the light‐electric co‐activated synapse responds to light and electric simultaneous stimuli in LECTS, leading to enhanced synaptic conductance contrast between light and dark states. By combining the unique capabilities of PDTFT and LECTS, we demonstrate their great potential for multimodal intelligent perception and high‐speed SNN supervised learning through achieving a high‐accuracy status detection strategy for autonomous vehicles and a smart navigation system for a walking robot. This work establishes a compact neuromorphic hardware paradigm that simultaneously enables millisecond‐level first‐spike tactile encoding and coupling‐enhanced synaptic learning, offering a high‐efficiency strategy for next‐generation spiking neural networks in autonomous perception and decision‐making systems.

## 4 Experimental Section

### Device Fabrication

### Device Fabrication—LECTS Fabrication

The LECTS (Light‐Electric Coupling Synaptic Transistor) was fabricated on an oxide wafer (Si_3_N_4_, 300 nm). The GaO_x_ precursor solution (McKinley, 99.99%) was spin‐coated at 3000 rpm for 30 s in a Class 100 cleanroom (relative humidity <30%). Annealing was performed in a muffle furnace (Thermo Scientific) under ambient air at 350 °C for 1 h. Subsequently, a 50 nm IGZO (In:Ga:Zn = 5:2:1, McKinley, 99.99%) channel layer was deposited at 4500 rpm for 50 s in three cycles via spin‐coating (precursor concentration: 0.2 mol L^−1^) and annealed at 370 °C for 1 h. Photolithography and wet etching were employed to pattern the IGZO channel. Source/drain electrodes (Au, 70 nm) were then deposited using ion‐beam evaporation, followed by annealing at 110 °C for 5 min.

### Device Fabrication—Dual‐TFT Fabrication

For the dual‐gate thin‐film transistor (Dual‐TFT), the process was similar to that of LECTS, except that it does not include the GaO_x_ layer. A 150 nm aluminum oxide (Al_2_O_3_) dielectric layer was deposited via magnetron sputtering under a metal shadow mask. The layer was annealed in air at 220 °C for 1 h. The top‐gate electrode (Au) was patterned using photolithography and lift‐off processes.

### Device Fabrication—Pressure Detection Sensor (PDs) Fabrication

The pressure‐sensitive layer was fabricated by dispersing multi‐walled carbon nanotubes (MWCNTs, Sigma‐Aldrich, 1.5 wt.%) into polydimethylsiloxane (PDMS, Dow Corning Sylgard 184) mixed with a curing agent at a 10:1 weight ratio. The mixture was degassed under vacuum for 2 min, spin‐coated onto a substrate, and thermally cured at 110 °C for 40 min. A 100 nm Au layer was then deposited on one side via thermal evaporation. Finally, the PDMS–Au composite film was cut into desired dimensions for integration.

### Device Fabrication—PDTFT Heterogeneous Integration

The pre‐fabricated piezoresistive film (PDs) was precisely aligned and laminated onto the top‐gate region of the dual‐gate IGZO TFT using a face‐to‐face contact process. The bonding was performed at room temperature with gentle pressure to ensure intimate physical contact while minimizing additional stress. The source and drain pad areas were kept exposed for subsequent electrical characterization and system integration.

### Measurement and Analysis

The electrical characteristics of dual‐TFTs and LECTS were measured using a semiconductor analyzer (Keithley 2636B) and a signal generator (Tektronix AFG31000). Memory behaviors were evaluated under illumination using a broad‐spectrum white LED light source (380–780 nm, peak wavelength ≈450 nm) at an intensity of 195 000 lux and under dark conditions (0 lux). The first spike data were collected through an NI signal acquisition card (USB‐6218) with multi‐channel MUX switching at a frequency of 500 Hz. The PDTFT and LECTS were characterized on separate probe stations and electrically connected through shielded wiring, as shown in Figure S27 (Supporting Information). The response, sensitivity, and durability of the piezoresistive thin‐film PDs were tested using a stepper motor (LinMot_Talk6) and a signal acquisition card. Ultraviolet Photoelectron Spectroscopy (UPS) was performed using the Thermo Fisher Scientific ESCALAB Qxi to determine the work function, and the device cross‐sectional structure was analyzed using a Thermo Scientific Helios 5 CX.

### Distance‐Dependent State Detection

Video processing experiments were conducted using 120 vehicular video samples (1.mp4–120.mp4) from a custom dataset. The YOLOv5s pretrained model (PyTorch implementation) was employed for vehicle detection with a confidence threshold of 0.85. Detected vehicle regions were converted to grayscale and resized to 125 × 125 pixels using OpenCV. The detailed method for pressure application and distance‐dependent state detection is provided in Note S8 (Supporting Information). In brief, the total EPSC was calculated as the normalized sum of all pixel intensities, while the average EPSC was derived only from the non‐zero (active) regions. Processed frames were recorded at 9‐frame intervals and stored in CSV files alongside synthesized video outputs (35 fps). The pipeline utilized Python libraries, including Torch, NumPy, and OpenCV, for spatial‐temporal feature extraction. The features, including total EPSC, average EPSC, and EPSC ratio, extracted from 120 samples across six states, were classified using a support vector machine (SVM).

### Spiking Neural Network Simulation

A 15×15 LIF neuron network model, derived from Note S6 (Supporting Information), was implemented using biological parameters: resting potential of 0 mV, threshold of 1 mV, membrane time constant of 10 ms, and a membrane resistance of 10 MΩ. The STDP learning rules were configured with A^+^ = 0.25, A^−^ = 0.05, τ^+^ = 10 ms, and τ‐ = 20 ms. Training consisted of 1500 frames (100 ms/frames) with input spikes coordinated across 2 × 2 neuronal blocks. Six target sequences received temporally‐coded inputs (t_1_‐t_4_ = 25–43 ms) while distractor blocks followed random patterns. Teacher signals reinforced correct temporal sequences through three‐phase weight updates: potentiation for silent target detection, depression for false positives, and neutral operation for validated patterns. To simulate the robot's movement along the planned path, the robot's feet were positioned within a 2 × 2 block, each containing a PDTFT/LECTS device. The pressure distribution in each block, which varies based on the robot's orientation and movement, encodes the interaction with its environment through FST sequences after PDTFT processing. The robot moves in a predefined direction and generates neuron spikes based on the sequence of detected pressure distributions.

For sequence detection, the system used spatiotemporal learning to recognize four different directional sequences. These sequences correspond to movement directions: up (29‐30‐14‐15), right (14‐29‐15‐30), down (15‐14‐30‐19), and left (30‐15‐29‐14). Each direction's sequence triggers a spike in the postsynaptic neuron. When the robot reaches a turning node, which corresponds to a target sequence, the postsynaptic neuron triggers a spike that stops the robot's movement and makes a directional decision. The robot continues its movement, following the highest EPSC path toward the next target sequence until the predefined path was completed. Finally, A path traversal test was conducted for 500 cycles to validate the robot's performance in traversing the planned path.

## Conflict of Interest

The authors declare no conflict of interest.

## Author Contributions

D.G., Y.W., and Y.J. supervised the project. D.G. and D.C. conceived the idea and designed the experiments. D.C. fabricated the PDTFT and LECTS devices, performed the electronic transport measurements, and analyzed the data. D.C. and F.Z. carried out the application‐level simulations of spiking neural networks (SNNs). M.S. and Y.L. conducted material characterization and data analysis. D.G., T.Z., and F.Z. wrote and revised the manuscript with input from all authors. All authors discussed the results and contributed to the final version of the manuscript.

## Supporting information



Supporting Information

## Data Availability

The data that support the findings of this study are available in the supplementary material of this article.
