# Supplementary material for: IGZO‐Based First Spike Timing Tactile Encoders and Coupling‐Enhanced Transistor Synapses for Efficient Spiking Neural Networks
Source: Adv Sci (Weinh). 2025 Dec 8;13(7):e11168. doi: 10.1002/advs.202511168 (PMC12866824; doi:10.1002/advs.202511168)
Supplement: Supplementary file 1 — Supporting Information [file ADVS-13-e11168-s001.pdf]

Supplementary Information

IGZO-Based First Spike Timing Tactile Encoders and Coupling-Enhanced Transistor Synapses for Efficient Spiking Neural Networks

*Dan Cai[1], Jinyong Wang[3], Tianchen Zhao[1], Miao Shen[1], Yunbo Liu[1], Tieyi Zhang[1], Fangjie Zhang[4], Yang Wang[1,2], Yadong Jiang[1,2], Deen Gu[1,2]**

[1]School of Optoelectronic Science and Engineering, University of Electronic Science and Technology of China, Chengdu 611731, PR China.

[2]State Key Laboratory of Electronic Thin Films and Integrated Devices, University of Electronic Science and Technology of China, Chengdu

[3]Department of Electrical and Computer Engineering, National University of Singapore, Singapore 117583, Singapore.

[4]Mianyang Huike Optoelectronic Technology Co., Ltd, Mianyang 621000, P. R. China.

*Corresponding author. Email: gudeen@uestc.edu.cn

Contents

Supplementary Notes 1 to 8

Supplementary Figure S1 to S27

Supplementary Table S1 to S3

Supplementary References

Note S1. The calculation of Sensor Sensitivity (S)

The sensor sensitivity (S) represents the slope of the relationship between the relative change in sensor resistance and applied stress, reflecting how sensitive the sensor is to mechanical signals (such as stress or pressure). It indicates the sensor's ability to detect small changes in mechanical signals. The higher the sensitivity, the easier the sensor can detect small mechanical signals.

$$S = \frac{\Delta R/R_0}{\Delta P} \quad (1)$$

Where Change in resistance (Δ R) is the amount by which the sensor's resistance changes under external stress. R_0 represents Initial resistance and Δ P represent the Change in stress[1]. Sensors with higher sensitivity can detect small mechanical signal changes more accurately, which is essential for applications requiring high-precision measurements, such as mechanical sensors and pressure sensors.

Note S2. The FST Encoding Mechanism of PDTFT

The top-gate input voltage U charges the parasitic capacitor C through the pressure-sensitive film (PDs).Since the PDs initially exhibit high resistance, when the resistance decreases to a certain extent, a significant voltage division effect forms between the PDs and the parasitic capacitor of the TFT.At this point, the external pressure-sensitive film resistance R(P) and the parasitic resistance Rc can be treated as equivalent to a parallel resistance Req. The maximum charging voltage can be expressed as:

$$R_{\mathrm{eq}} = \frac{R(P)R_c}{R(P) + R_c}, V_{\max} = \frac{UR_c}{R(P) + R_c} \quad (2)$$

The time t(P) required for the capacitor to charge to the trigger voltage U_{target} (i.e., Sub_Vth) is given by:

$$t(P) = -R_{\mathrm{eq}}C\ln\left(1 - \frac{U_{\mathrm{target}}}{V_{\mathrm{max}}}\right) \qquad (3)$$

IGZO TFT Triggering Resting Condition

The condition for proper charging to achieve a resting period is:

$$0 < U_{\mathrm{target}} < V_{max} = \frac{UR_c}{R(P) + R_c} \qquad (4)$$

If this condition is not met, $t(P)$ will either continuously trigger or never trigger, making it impossible to realize FST encoding. Therefore, V_{max} determines whether the device has a controllable operating range.

Since the device exhibits a linear response within the pressure range of P∈[0,1.54] kPa (Figure 3b), its resistance can be expressed as:

$$R(P) = R_0(1 - S)P \qquad (5)$$

Here, S represents the sensitivity, with S=67.31kPa. Since V_{max} depends on the relative magnitude of the initial resistance R_0 and the parallel resistance R_{c} it determines whether the device possesses a tunable operating range. For clarity, three typical cases will be discussed below based on the relationship between R_0 and R_{c} .

（1） Low-Resistance Region ($R_0 \ll R_c$)

$$R_{eq} \approx R(P), V_{max} \approx U \qquad (6)$$

$$t(P) \approx -R(P)C\ln\left(1 - \frac{U_{target}}{U}\right) \qquad (7)$$

In this case, t(P) has an approximately linear relationship with the pressure P. Our experimental results confirm this characteristic, as shown in Figure S6, t(P) changes linearly

with P, indicating that the initial resistance of the PDs is much lower than the equivalent resistance corresponding to the TFT parasitic capacitor.

（2） Medium-Resistance Region ($R_0 \sim R_c$)

When R(P) and Rc are comparable, the voltage division effect is significant, and the first-spike delay is:

$$t(P) = -\frac{R(P)R_c}{R(P)+R_c} C \ln \left(1 - \frac{U_{target}}{U \cdot \frac{R_c}{R(P)+R_c}} \right) \quad (8)$$

In this case, t(P) exhibits a nonlinear response as the pressure P increases.。

（3） High-Resistance Region ($R_0 \gg R_c$)

When $R(P) \gg R_c$, most of the voltage drop occurs across the PDs, which can be approximated as:

$$R_{eq} \approx R_c, V_{max} \approx U \cdot \frac{R_c}{R(P)} \ll U \quad (9)$$

$$t(P) \approx -R_c C \ln \left(1 - \frac{U_{target}}{V_{max}} \right) \quad (10)$$

If $U_{target} \geq V_{max}$,, the capacitor voltage can never reach the threshold, meaning the device remains permanently in the resting state without triggering.`

Note S3. Crosstalk Simulation of the Array

The pressure-sensitive film (PDs) forms a continuous resistive layer across the array. When a single pixel is loaded, mechanical deformation propagates outward, altering the resistance of neighboring pixels and causing voltage variations during RC charging—manifesting as crosstalk.

A 4×4 finite element model (pixel pitch: 4 mm, electrode gap: 1.5 mm, total area: 23×23 mm², Figure S9) was built in COMSOL, with each PDTFT represented as a series RC unit. Coupled solid mechanics and electric current modules were used: the mechanics module works out the stress and strain distributions under localized pressure, while the current module analyzes the voltage-current relationships of the device as on the resistance of the PD changes. The equivalent strain is defined as:

$$\varepsilon_{\mathrm{eq}} = \sqrt{\frac{2}{3}\left(\varepsilon_{xx}^2 + \varepsilon_{yy}^2 + \varepsilon_{zz}^2 + 2\varepsilon_{xy}^2 + 2\varepsilon_{yz}^2 + 2\varepsilon_{xz}^2\right)} \quad (11)$$

Based on the fitting of the experimental data of P versus $\Delta R/R_0$ shown in Figure 3b, and through model derivation, the conductivity is found to exhibit an exponential relationship with strain:

$$\sigma = \sigma_0 \cdot \exp\left(\alpha \cdot \varepsilon_{eq}\right) \quad (12)$$

Here, σ_0 is the initial conductivity of the PDs (3.6×10^{-7} S/m) , and α is the fitting parameter with a value of 80, as shown in the calculation process in Figure S10.

In the calculation of crosstalk, the target stressed pixel is defined as the main pixel region (Ωmain), while the other neighboring affected pixels are defined as crosstalk pixel regions (Ωi). The current density distribution is obtained from the Eq. (12), and the total current of each region is calculated through surface integration:

$$I_{main} = \iint_{\Omega_{main}} \sigma * E \, dS \quad (13)$$

$$I_{\text{crosstalk}} = \sum_{i=1}^{4} \iint_{\Omega_i} \sigma * E\, dS \tag{14}$$

Crosstalk was quantified as the current ratio of adjacent pixels to the loaded pixel:

$$\text{Crosstalk} = \frac{I_{\text{crosstalk}}}{I_{\text{main}}} \times 100\% \tag{15}$$

Figure S11 shows the stress and current distributions of the PDs under different pressures (1, 50, 75, and 100 kPa). It can be observed that at a low applied pressure (1 kPa), the stress concentration region is confined directly beneath the loaded pixel with a very small magnitude, and its influence on the surrounding area is almost negligible. When the pressure increases to 50 kPa, the stress beneath the target pixel intensifies significantly and begins to diffuse laterally along the film plane, affecting adjacent pixel regions. As the pressure further increases to 75 kPa and 100 kPa, the stress peak continues to rise, forming distinct concentric diffusion rings. This indicates that external mechanical disturbances are transmitted through the continuous coupling of the film, thereby inducing cooperative deformation in neighboring pixels.

Figure S12 presents the finite element simulation results of the current density at the top electrode of PDs under different pressures (1, 50, and 100 kPa). From the cross-sectional view, it can be seen that at 1 kPa, the current distribution is uniform, with only a slight enhancement at the loaded pixel and negligible influence on surrounding units. At 50 kPa, the current density of the target pixel increases significantly and diffuses outward, but there is still a noticeable decay at the boundaries. At 100 kPa, the peak value further increases and induces signals at the edges of adjacent pixels, while no obvious current coupling effect is observed.

Figure S13 also presents the quantitative relationship between crosstalk level and pixel spacing (expressed as Ic/Imain). Crosstalk decayed rapidly with distance, following a power-law

trend (Ic/Imain ∝ d−n（n≈1~2）). At a 1.5 mm pitch, the crosstalk was only 0.61%, far below the typical engineering tolerance of 1–2%, confirming that even under maximum load, electrical crosstalk remains negligible. Therefore, a 1.5 mm pitch is validated as a safe design rule.

Note S4. LTD behavior and mechanism of LECTS under light and without light

In Figures S15, the device exhibited different response currents under the three stimulation modes with positive BG bias, which differs from the behavior under negative bias. When voltage pulses were applied solely to the transistor gate, the output current showed a conductance reduction, indicating the device exhibited LTD in this mode. Under continuous light exposure with positive bias, the device exhibited the highest PSC. This confirms that the light-induced effect weakens LTD.

The memory behavior also can be explained by band theory analysis and dynamic process of carrier migration. When a positive voltage was applied to the BG without light, the positive gate voltage caused electrons in the IGZO to migrate toward the GaOx and Si_3N_4 layers. Meanwhile, the GaOx/IGZO heterojunction (GaOx: WF = 4.58 eV / IGZO: WF = 4.08 eV, as shown in Figure 4e) was offset by the external electric field, leading to a reduction in the energy band barrier at the interface. As a result, electrons left the IGZO channel, exhibiting LTD behavior. When the device is exposed to light, electron-hole pairs are generated in the GaOx and IGZO layers. Under the influence of both the internal electric fields at the heterojunction and the external electric field (Vgs), the carriers are separated, resulting in an increased number of electrons. This increase in electron injection compensates for the loss of electrons migrating from the IGZO channel, which leads to a reduction in the LTD effect under light exposure.

Note S5. KPFM Method

KPFM measurements were performed using an atomic force microscope (Bruker Dimension Icon) in AM-KPFM mode. The sample consisted of an IGZO film partially covered with GaO_x (Figure S18). The scan path crossed the GaO_x/IGZO interface to obtain the surface potential distribution in the interfacial region. During testing, the bottom of the sample substrate was grounded, and a conductive probe (Pt/Ir-coated Si, with a tip radius of approximately 20–30 nm) was biased with a combination of AC and DC voltages::

$$V(t) = V_{\mathrm{DC}} + V_{\mathrm{AC}} \sin\ \omega t \qquad (16)$$

The Kelvin controller automatically adjusted V_{DC} to nullify the first harmonic electrostatic force signal, at which point V_{DC} corresponds to the contact potential difference (V_{CPD}) between the probe and the sample. Measurements were carried out under dark conditions and various illumination intensities, with uniform light exposure over the scanned area.

According to the KPFM principle, the probe work function Φ_{tip} was calibrated using a reference sample with a known work function, and the sample work function was calculated using the following equation:

$$\Phi_{\mathrm{s}} = \Phi_{\mathrm{tip}} - eV_{\mathrm{CPD}} \qquad (17)$$

Thus, with a fixed Φ_{tip}, a larger V_{CPD} indicates a smaller sample work function Φ_{s}, corresponding to a higher Fermi level (closer to the conduction band).

Note S6. LIF Post-Neuron Model

The Leaky Integrate-and-Fire (LIF) neuron model is a simple yet effective model used to simulate the dynamics of spiking neurons in computational neuroscience. It incorporates membrane potential dynamics, synaptic input, and threshold-based firing behavior.

In the LIF neuron model, the membrane potential V(t) evolves over time according to the following differential equation[2]:

$$\tau \frac{dV}{dt} = -(V - V_{\text{rest}}) + R \cdot I(t) \qquad (18)$$

Where τ is the membrane time constant, controlling the rate of membrane potential decay towards its resting potential V_{rest}. R is the membrane resistance, $I(t)$ is the input current at time t. The detail parameters can be found in method section. The model incorporates a leakage term which accounts for the natural decay of the membrane potential toward its resting state, and a synaptic input term that increases the membrane potential based on incoming current.

When the membrane potential V exceeds the firing threshold Vth, the neuron emits a spike. Upon spike detection, the neuron's membrane potential is reset to its resting value Vrest , and a spike duration counter is initiated to control the refractory period. During this period, the neuron remains "inactive" despite further input. The output spike is represented by a binary signal:1).1 if the neuron fires a spike (when V ≥ Vth).2). 0, otherwise.

This model is widely used to simulate spiking neural networks (SNNs), where the precise timing of spikes is crucial for encoding information. The behavior of LIF neurons provides a good approximation of the dynamics found in biological neurons while remaining computationally efficient for large-scale simulations.

Note 7: Definition of Training Time and Convergence Criteria in SNNs

In our spatiotemporal learning task, each training step corresponds to the input of one frame lasting 100 ms, which contains four sequential spikes from a 2×2 sensory block (e.g., units 77 → 78 → 62 → 63). Thus, the total training time is defined as: Training time = number of frames × 100 ms. Each complete run consists of 1500 frames, equivalent to 150,000 ms (150 s) of simulated time. Convergence is reached when all target postsynaptic neurons are correctly activated and no non-target outputs fire for at least 10 consecutive frames. A firing threshold of 1 V is applied: neurons fire only when their membrane potential exceeds this threshold, while non-target synapses remain fully suppressed. The baseline SNN requires 110 frames to reach convergence, including time to reinforce target synapses and suppress non-target synapses. With the light-focused training strategy, non-target suppression is greatly accelerated, reducing the convergence time to only 10 frames, a 90.9% decrease (110 → 10 frames), averaged over five independent runs under identical conditions.

Note 8: Detailed Method for Pressure Application and Distance-Dependent State Detection

To clearly illustrate how the six states in Figure 5e are generated, we provide a detailed explanation of the pressure application method and its relationship to the distance-dependent signal variations. These six states are determined by the combined encoding of PDTFT pressure signals and LECTS light–electric coupling synapses, which jointly capture both motion state and relative distance.

The classification is based on two independent features:

1.EPSC Ratio – reflects the relative distance between vehicles, distinguishing three distance scenarios: Far, Mid, and Near.

2.Average EPSC – represents the motion state of the current vehicle, distinguishing two scenarios: Idle and Moving.

By combining these two factors, the system distinguishes six unique states (S1–S6), as summarized in Table S2.

1. Determining Relative Distance (EPSC Ratio)

The relative distance is determined by the size of the illuminated area on the 125 × 125 LECTS array. A larger illuminated region corresponds to a closer lead vehicle, while a smaller region represents a farther distance. This relationship is quantitatively captured by the EPSC Ratio:

$$EPSC\ Ratio = \frac{\sum_{i=1}^{n} (S \times PixelValue_i)_{final}}{\sum_{i=1}^{n} (S \times PixelValue_i)_{initial}} \quad (19)$$

Here, n=125×125 is the total pixel count. The terms "initial" and "final" refer to the first and last frames of the observation window. EPSC Ratio ≈ 1 → Constant distance; EPSC Ratio > 1 → Lead vehicle is approaching; EPSC Ratio < 1 → Lead vehicle is receding. This feature compares the temporal change of EPSC values to detect dynamic distance variations.

2. Detecting Motion State (Average EPSC)

The motion state of the current vehicle is determined by the pressure applied to the PDTFT. Higher pressure produces earlier PDTFT spikes, which result in larger EPSC amplitudes. The Average EPSC is calculated as:

$$\text{Average EPSC} = \frac{\sum_{i=1}^{N_{\text{non-zero}}} \left(S \times \text{Pixel\_Value}_i\right)}{225 \times N_{\text{non-zero}}} \quad (20)$$

Where:$N_{\text{non-zero}}$= total number of active pixels in the illuminated region.Pixel_Value_i= gray level of the ith pixel (0–225).Scaling factor S: Idle = 1.73 nA, Moving = 15 nA. Threshold ranges: Idle state → Average EPSC ≤ 10^{-9} A; Moving state → Average EPSC ≥ 10^{-8} A. Thus, by monitoring pressure-induced EPSC levels, the PDTFT can accurately determine whether the vehicle is Idle or

Moving. These two independent features provide clear and non-overlapping numerical thresholds for state identification (Table S3).

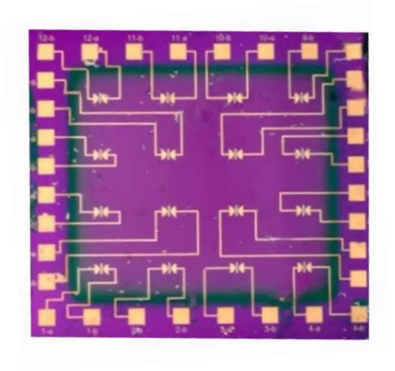

Figure S1. Optical image of the dual TFTs in a 4x4 array overview.

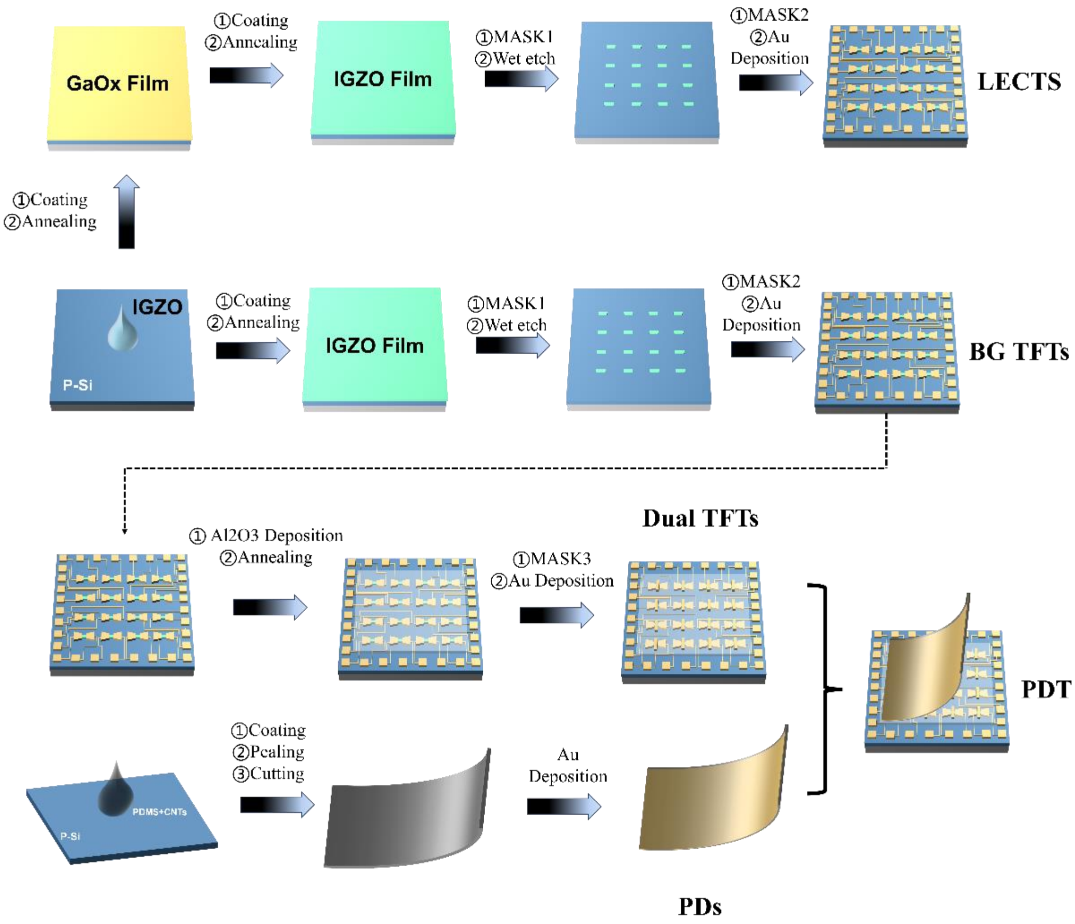

Figure S2. Schematic diagram of the fabrication process for PDT and LECTS devices. Detailed process parameters are referenced in the Method section.

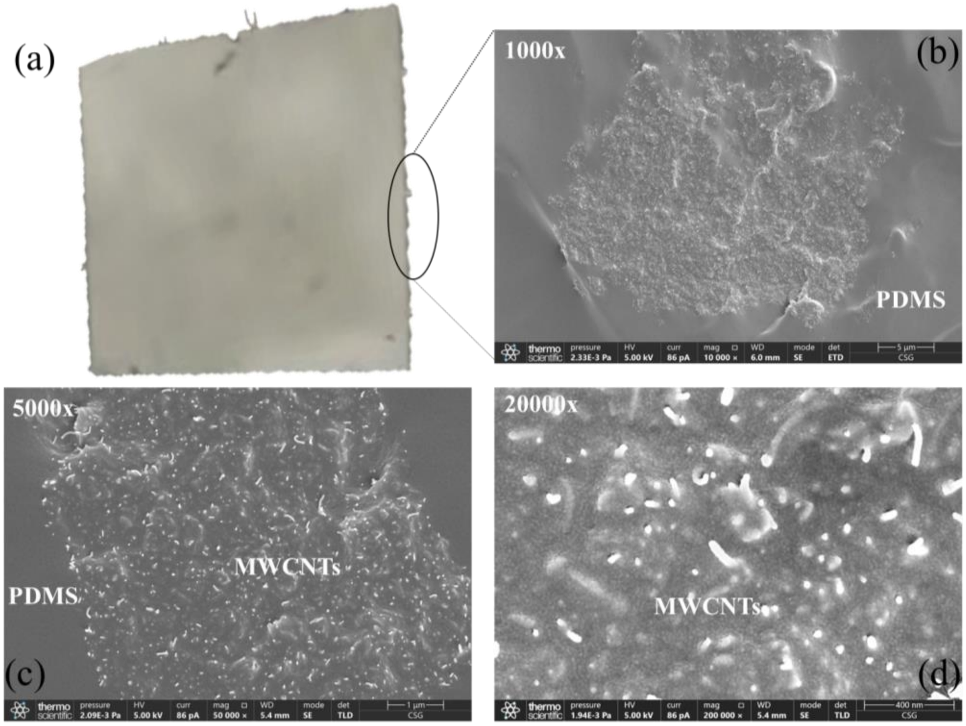

Figure S3. SEM images of the PDMS/MWCNT interface at different magnifications

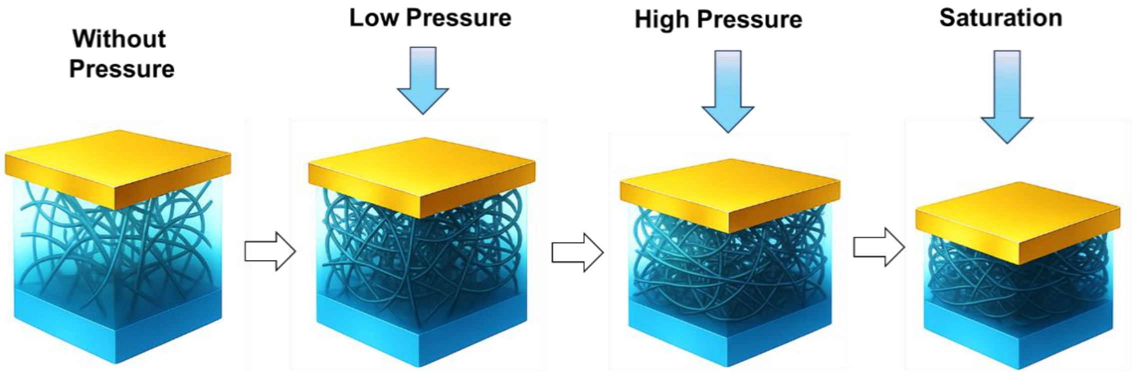

Figure S4. Schematic illustration of the pressure-sensitive mechanism of the PDMS/MWCNT composite.

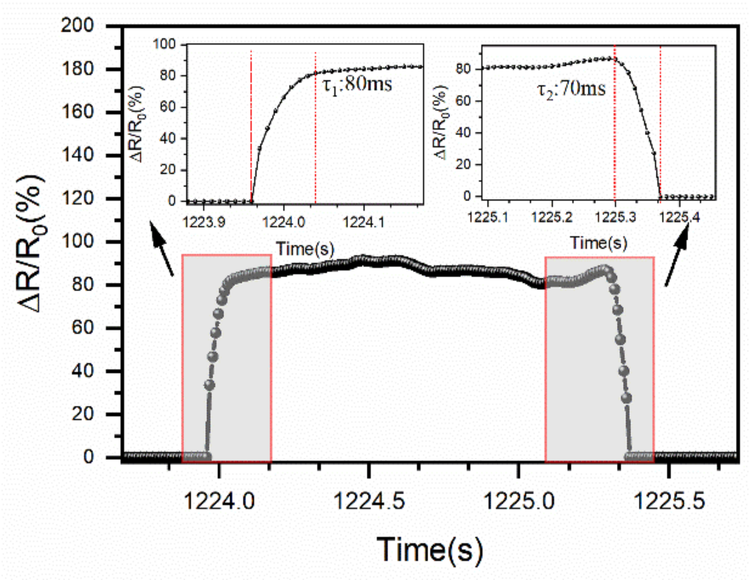

Figure S5. The response time and recovery time of PDs during a pressure test at 35 kPa^{-1} and a frequency of 0.33 Hz.

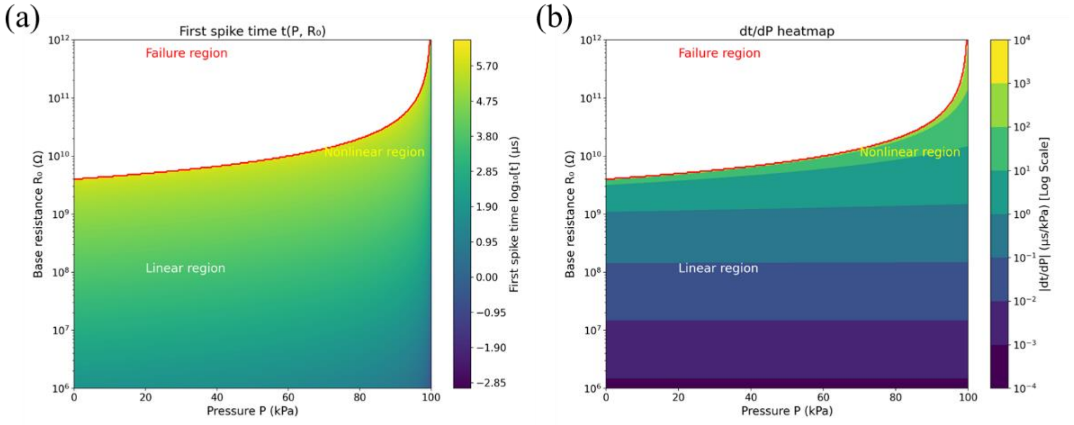

Figure S6. Effect of the initial resistance R_0 of PDs on device response. (a) Distribution of first-spike triggering time t(P,R0) and working region division; (b) distribution of pressure sensitivity dt/dP, with the red line indicating the boundary between triggering and failure.

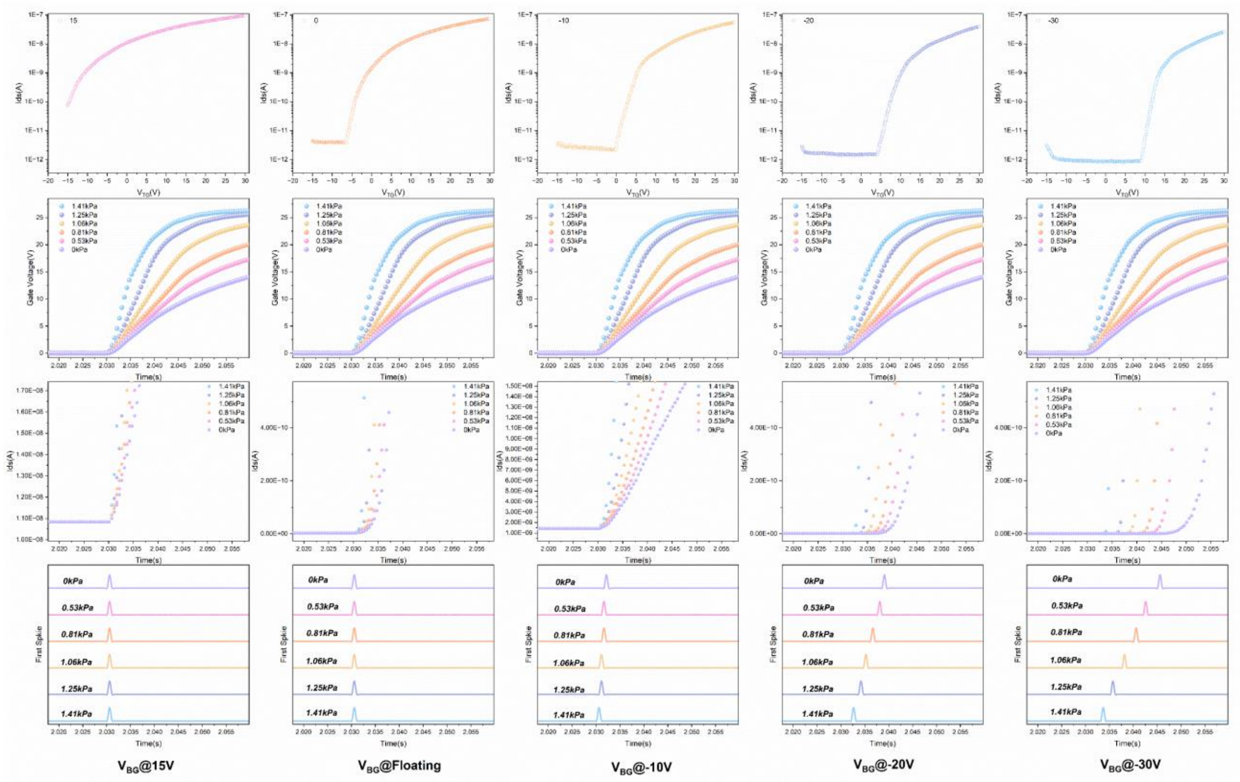

Figure S7. The entire process of PDTFT under BG modulation at-30V, -20V, -10V, 0V and 15V, respectively. The first row shows the ID-VG curves; the second row presents the V-T charging curves on the TG at various pressures; the third row shows the drain current (Ids) as the charging voltage increases; the fourth row depicts the first spike generation during TG charging, based on the Ids in the third row.

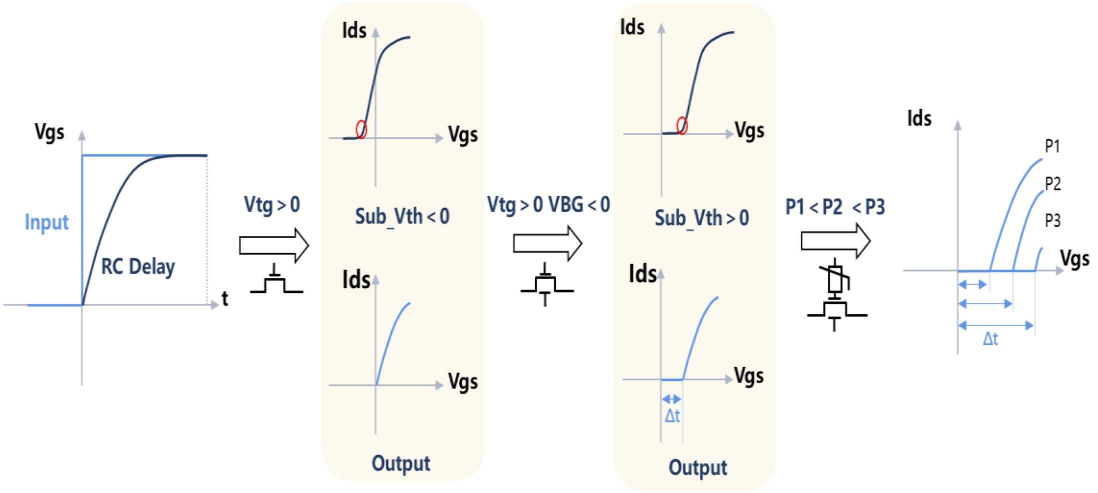

Figure S8. The generation process of the resting state is modulated by the subthreshold voltage (Sub_Vth) of the Dual-TFTs.

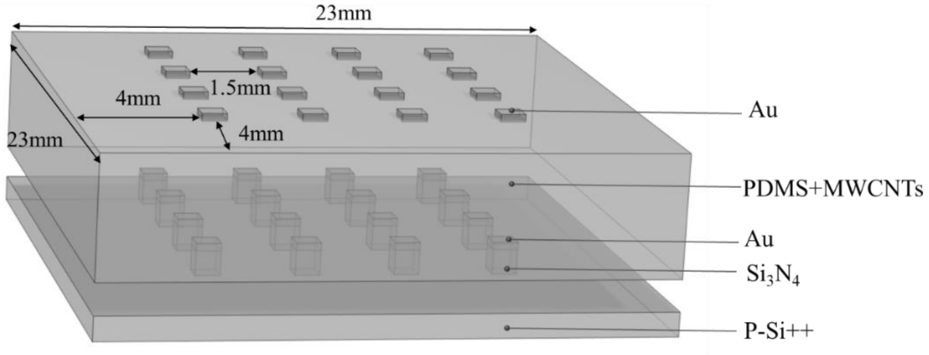

Figure S9. Geometrical structure of the 4×4 PDTFT array

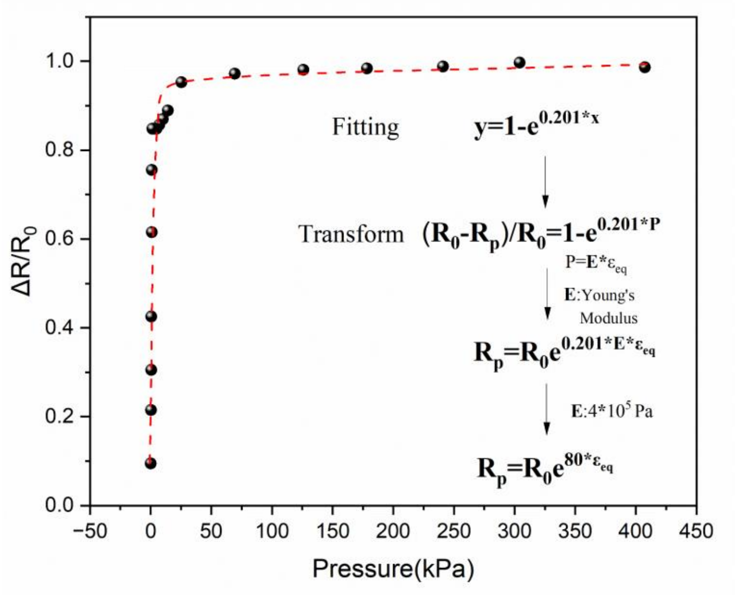

Figure S10. Fitting of the stress–conductivity relationship of PDs

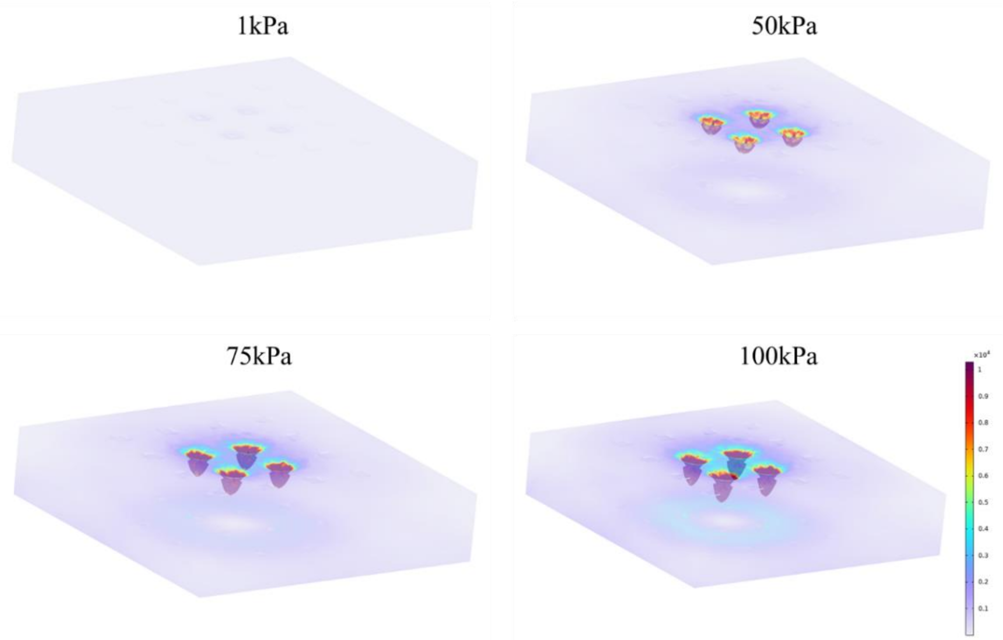

Figure S11. Finite element simulation of PDs stress under different pressures.

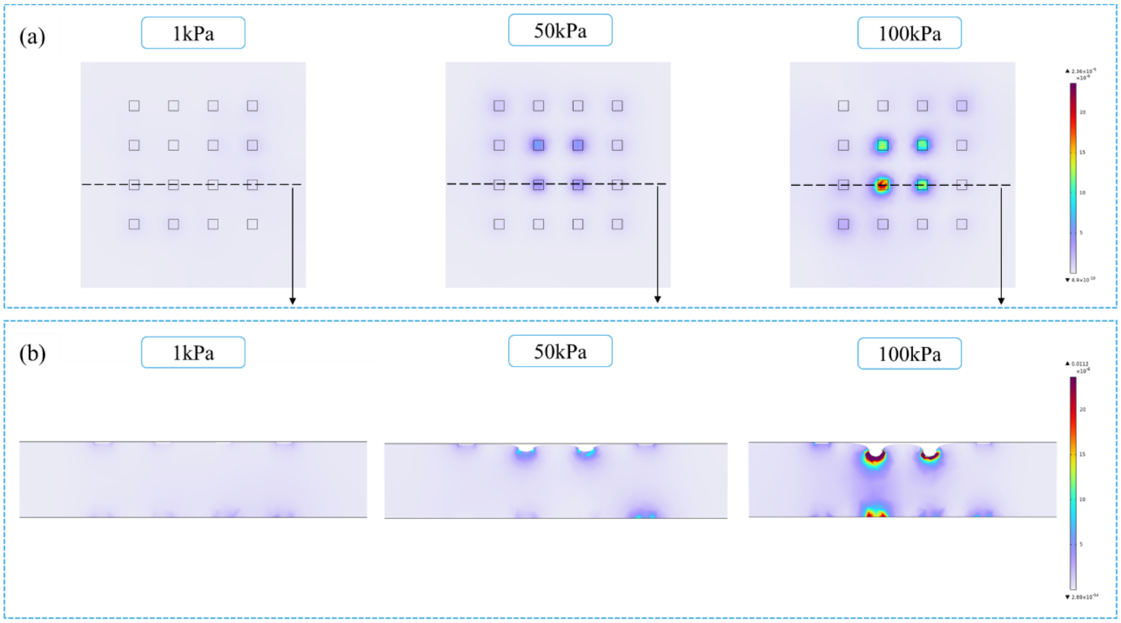

Figure S12.Finite element simulation results of the output current density at the top electrode of the PDs under different pressures. (a) Top view; (b) Cross-sectional distribution along the dashed line.

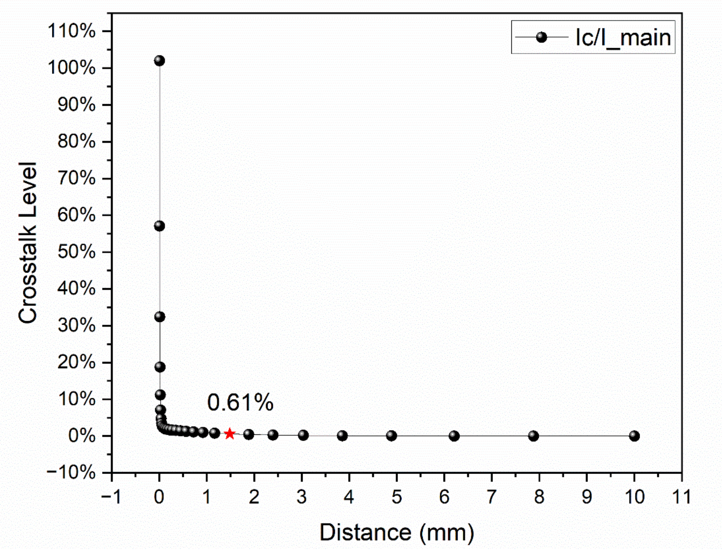

Figure S13. Correlation between device spacing and crosstalk level.

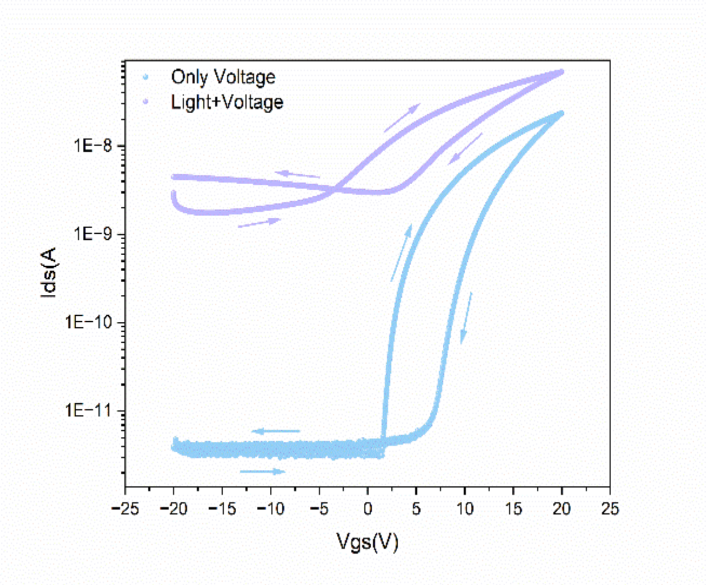

Figure S14. Hysteresis loop of ID-VG for LECTS under light and in the absence of light. It illustrates that when Vgs is positive, a memory window exists, and the conductance decreases, showing LTD even without light. When Vgs is negative, there is no memory window; however, with the introduction of light, a distinct conductance enhancement memory window appears, accompanied by a higher EPSC.

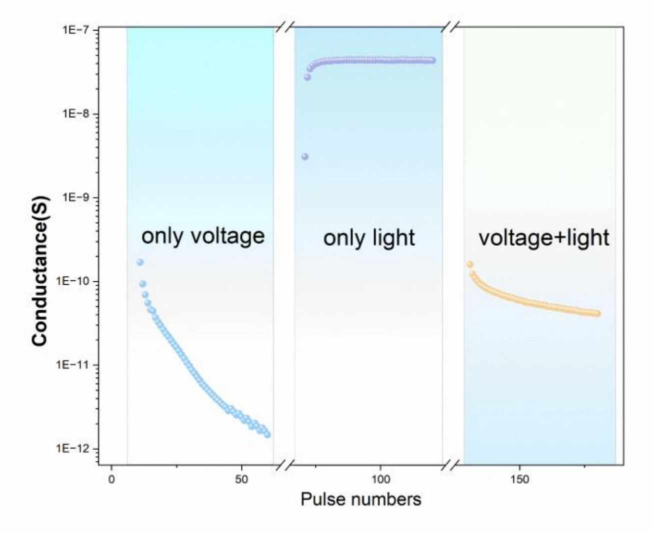

Figure S15. The three stimulation modes applied to the bottom gate with positive bias of LECTS: light-only, electrical-only, and light-electric coupling responses.

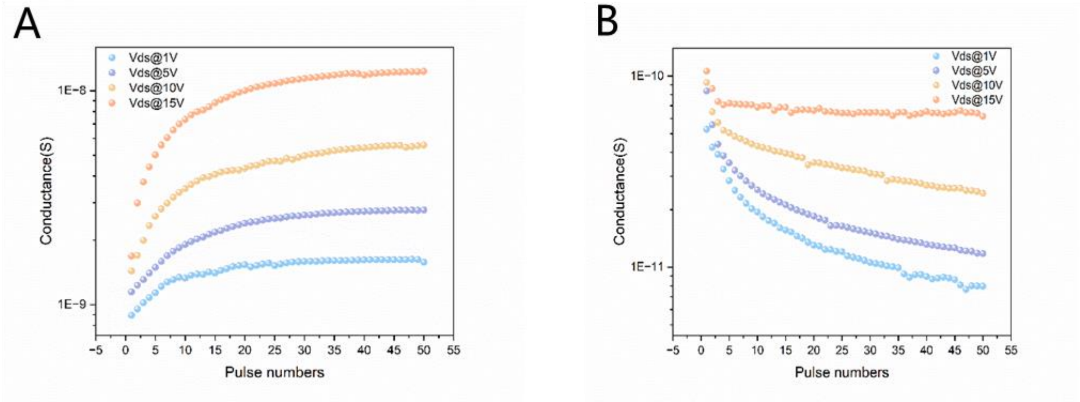

Figure S16. The performance of memory behavior under various Vds. (A) Applied 50 voltage pulses [-30V, 0.5s] on the BG for LTP, with a read voltage of [-0.2V, 0.1s]. (B) Applied 50 voltage pulses [30V, 0.5s] on the BG for LTD, with a read voltage of [-0.2V, 0.1s].

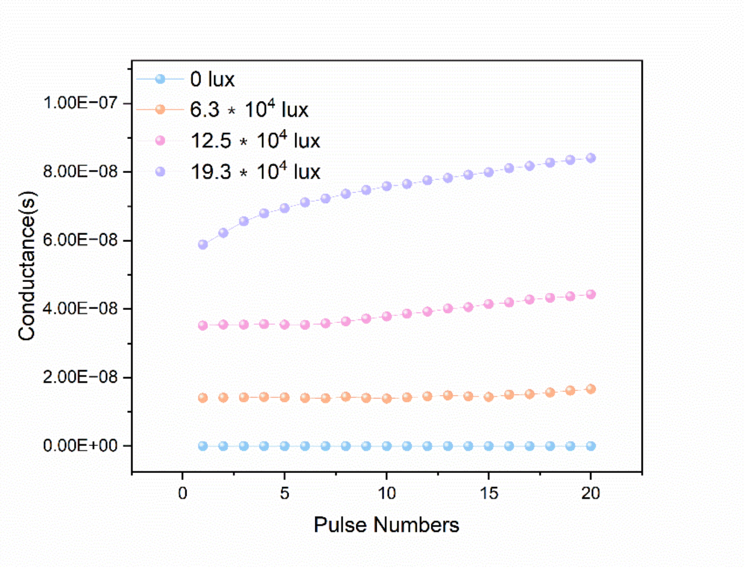

Figure S17. Conductance evolution under multiple electrical pulses under illumination at a fixed gate voltage of −30 V, with read voltage [0.2V.0.1s]

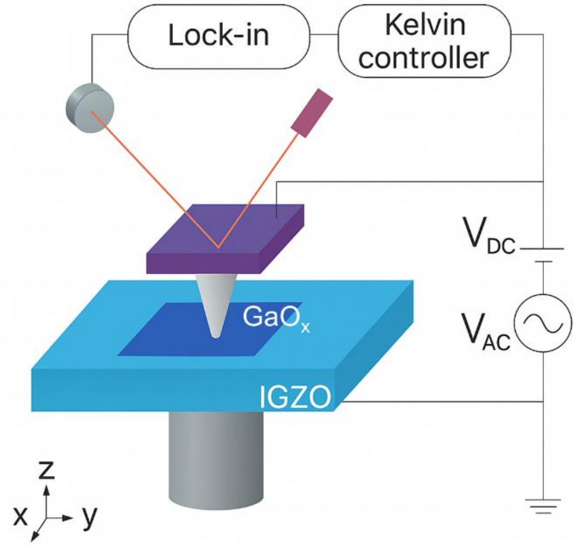

Figure S18. Schematic illustration of the KPFM potential measurement principle.

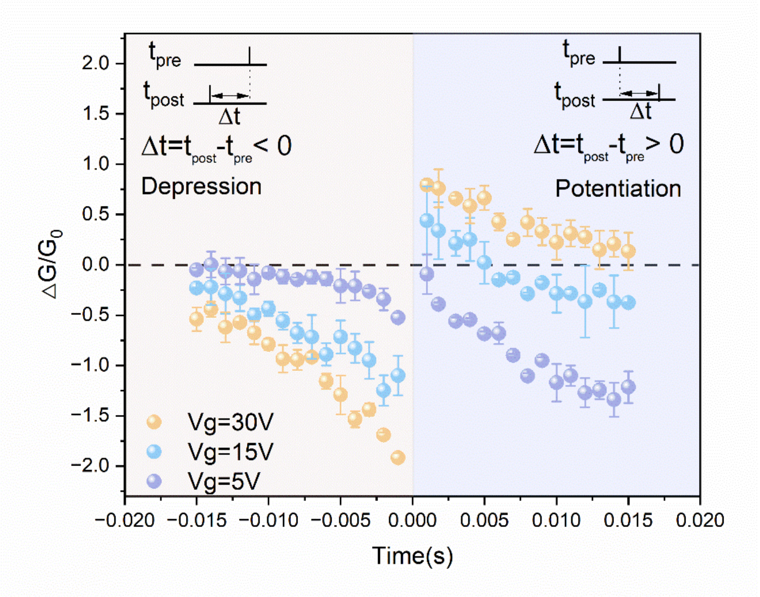

Figure S19. STDP of LECTS under dark condition with different gate voltages

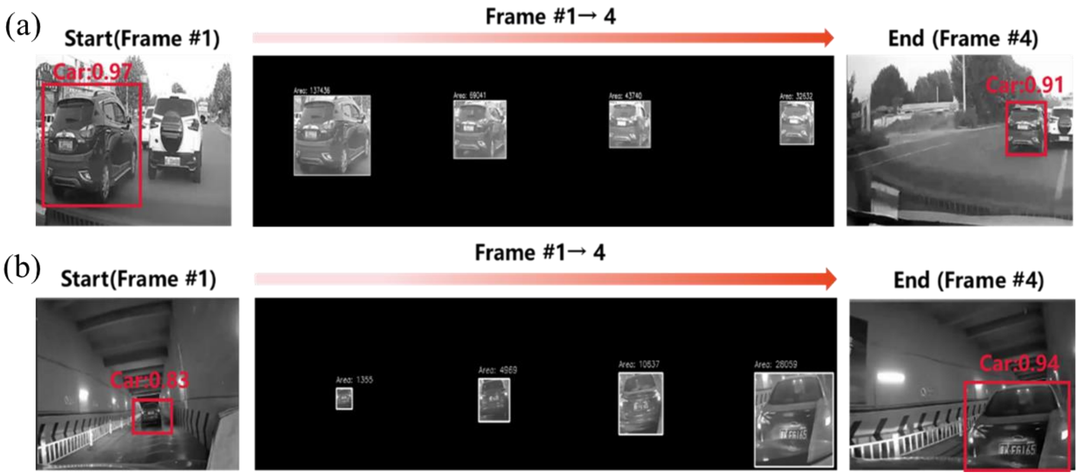

Figure S20. The relative distance between the front and rear vehicles during the driving process. (A) When the speed of the front vehicle is greater than that of the rear vehicle, the relative distance increases and the image size decreases, indicating the 'Overtaken' state. (B) When the rear vehicle is in the 'Idle' state, the relative distance decreases and the image size increases, indicating the 'Roll-Back' state.

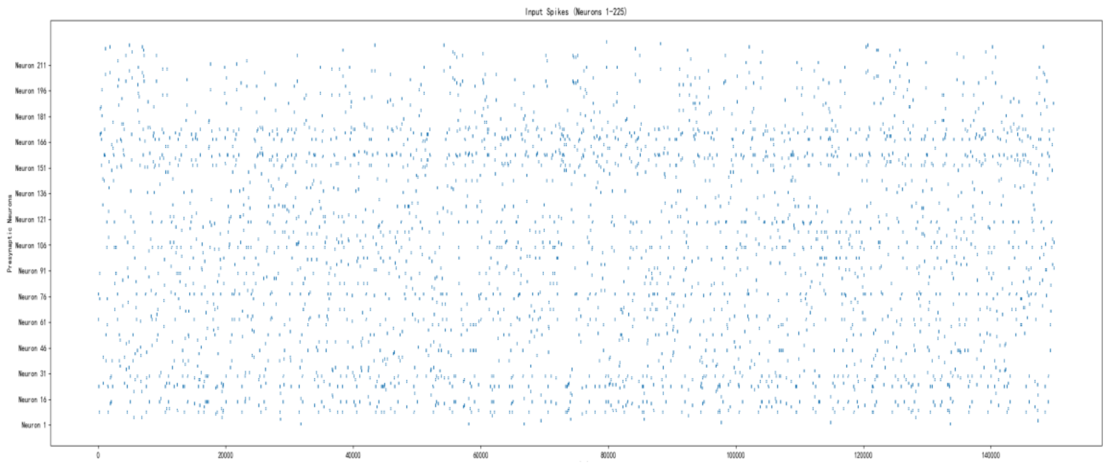

Figure S21. Pre-neuron input spikes in SNN training. A random 2x2 block is selected from the 15x15 synapse array. These blocks consist of two parts: (1) along the planned path, where the robot’s pressure distribution is associated with first spike timing (FST encoding) between 25 and 43 ms, and (2) off the path, where no pressure is present and all spikes occur at 43 ms. Each frame contains 4 FST-encoded spikes.

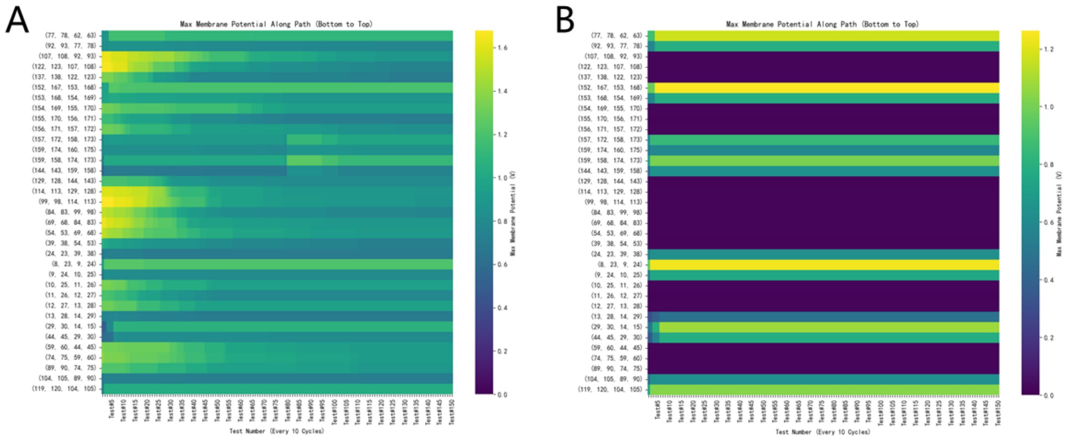

Figure S22. The Max EPSC of blocks aligned with the designed path during synapse updates every 100 cycles. (A) Without focused light. (B) With focused light.

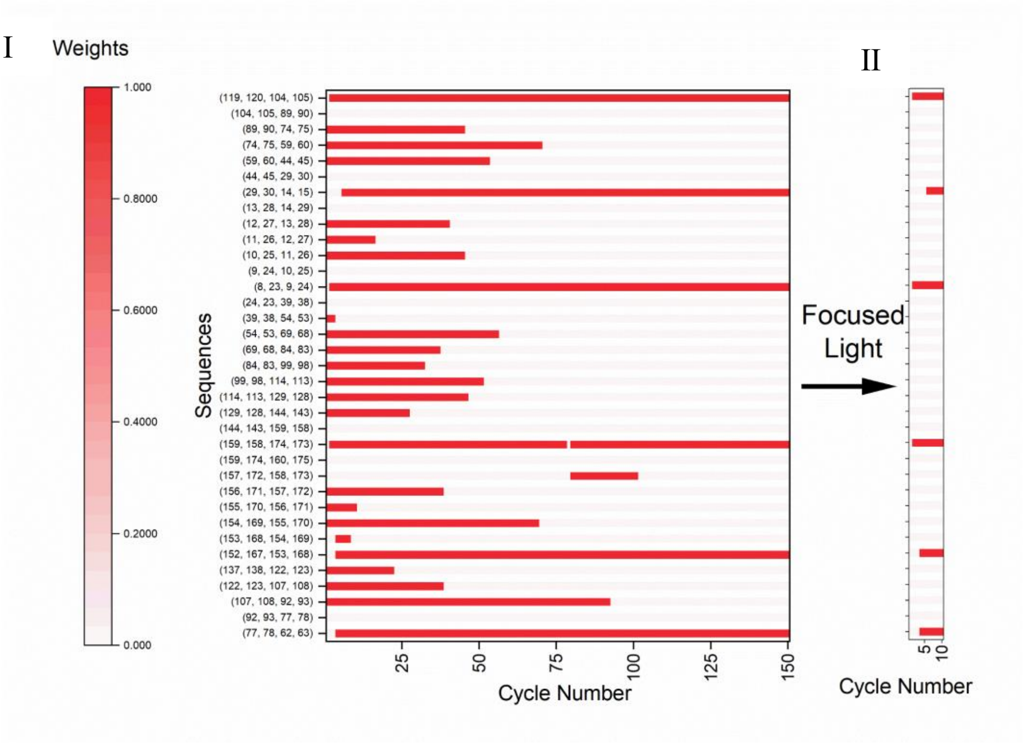

Figure S23. Neuron firing behavior of 2×2 blocks along the designed path during training (cycles per 10 frames), under conditions without focused light (I) and with focused light (II).

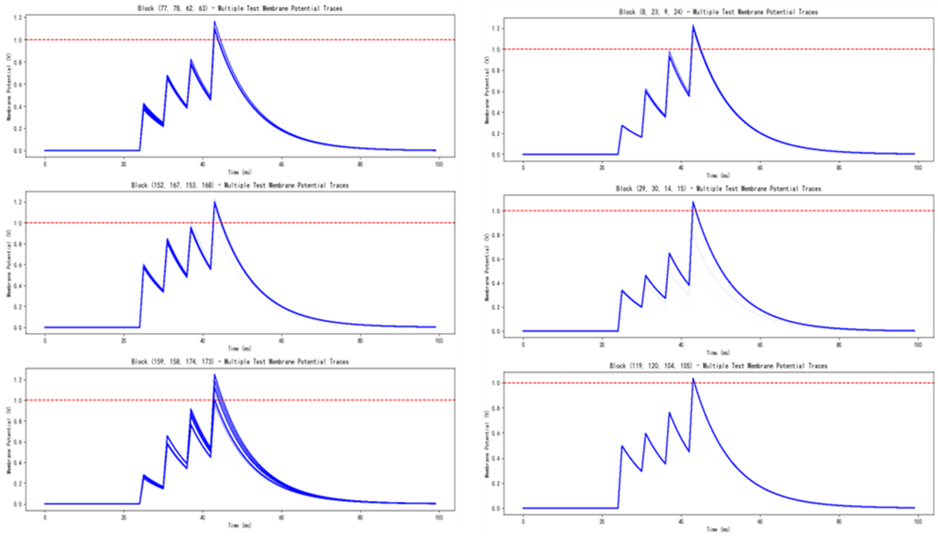

Figure S24. The maximum EPSC of six randomly selected blocks (4 spikes) aligned with the designed path under the final synapse weight during the continuous update process. It can be observed that with ongoing weight updates, the maximum potential of the post-neuron gradually approaches the threshold voltage (Vth = 1.0V), triggering a spike.

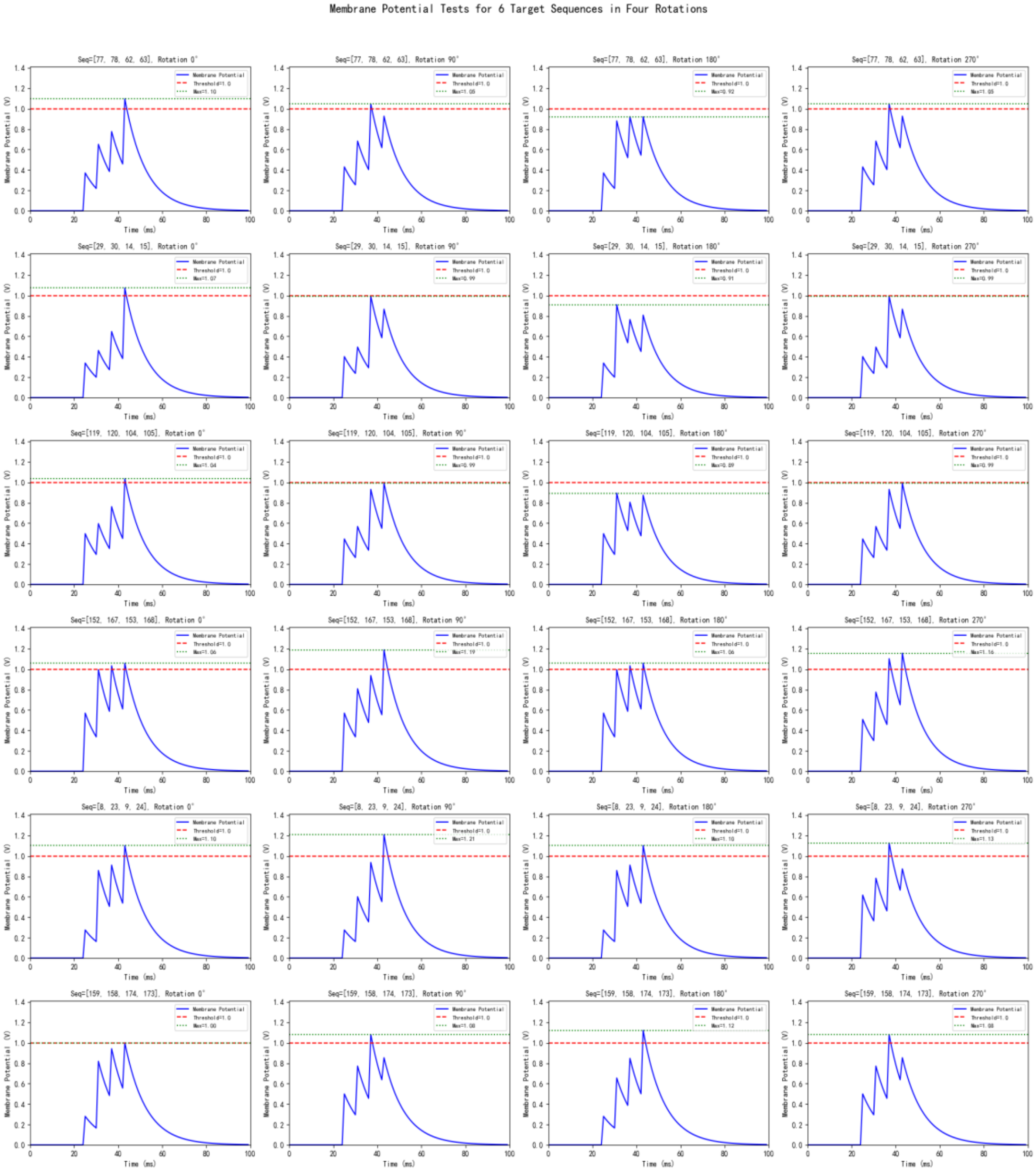

Figure S25. The four directions of the robot stand in 2x2 blocks, which act as turning nodes. The input spikes are weighted and amplified by the synaptic weights, then processed through the post-

neuron using LIF computation. In each block, regardless of the sequence, the post-neuron may fire. However, only when the target direction corresponds to the maximum EPSC does the post-neuron trigger.

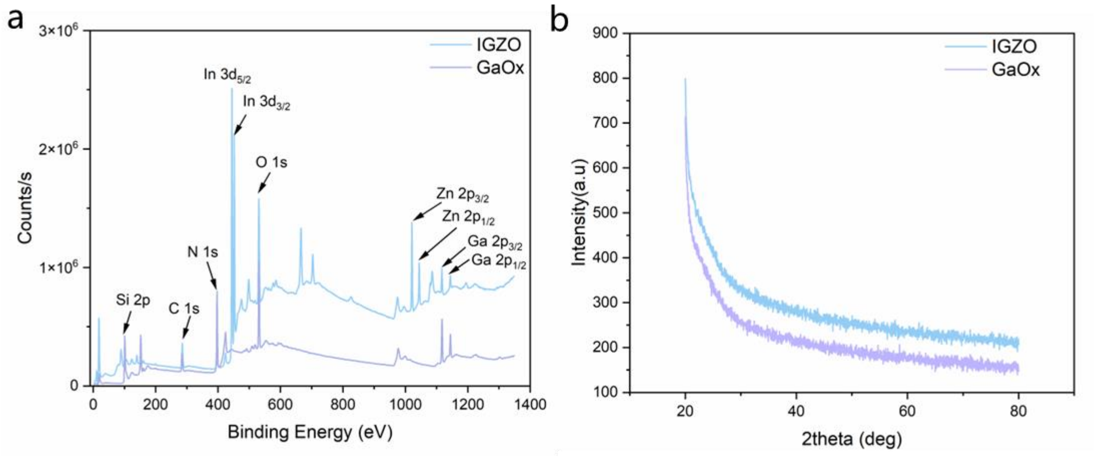

Figure S26. (a) XPS survey spectra of IGZO and GaOx.(b) XRD patterns of IGZO and GaOx.

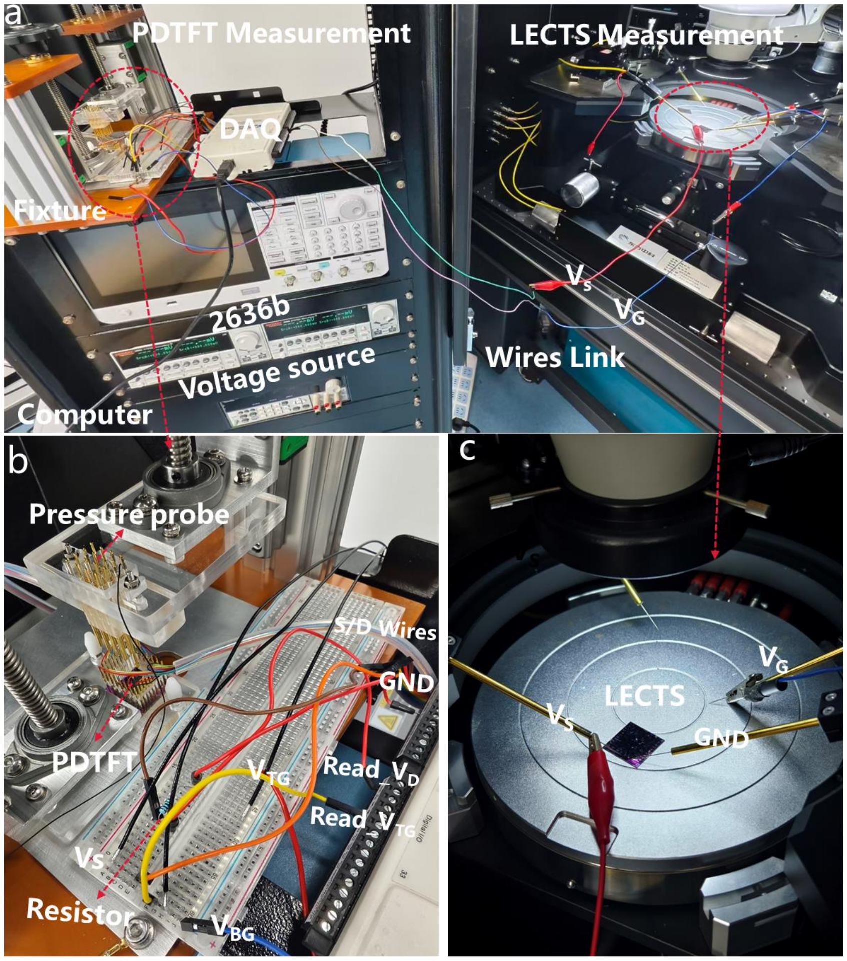

Figure S27. Actual wiring and measurement setup for PDTFT-to-LECTS signal transmission. In this setup, the PDTFT and LECTS were measured on separate probe stations and electrically connected through wires. The PDTFT was driven by three independent terminals (source, top gate, and back gate), and its current output was sampled by the DAQ module. Based on the extracted first-spike timing, the DAQ generated FST voltage pulses and precisely controlled the time interval (ΔT) between the gate and source pulses. Under LED illumination, the ΔT-dependent gate excitation modulated the channel conductance of the LECTS, enabling synaptic plasticity.

Table S1. Comparison of the Current State of Spike Timing Encoding Research

Encoding mechanism	Encoding scheme	Year	Ref.
Inter-spike Interval Encoding	Software	2018	[3]
Inter-spike Interval Encoding	Software	2018	[4]
Inter-spike Interval Encoding	Software	2020	[5]
Inter-spike Interval Encoding	Software	2021	[6]
First spike Timing Encoding	Circuits	2022	[7]
Spike Train Duration Encoding	Circuits	2020	[8]
Spike Train Duration Encoding	Circuits	2024	[9]
First spike Timing Encoding	Circuits	2024	[10]
Spike Train Duration Encoding	Device	2021	[11]
First spike Timing Encoding	Device	2025	This work

Table S2. Classification of six driving states based on Average EPSC and EPSC Ratio

Average EPSC / EPSC Ratio	Idle (No Pressure)	Moving (With Pressure)
Far (Small Area)	S1_Start	S2_Overtaken
Mid (Medium Area)	S3_Stop	S4_Fellow
Near (Large Area)	S5_Accelerate	S6_Roll-Back

Table S3. Quantitative criteria for six driving states based on PDTFT motion input and LECTS distance input

State	PDTFT Input (Motion)	LECTS Input (Distance)	Average EPSC	EPSC Ratio
S1_Start	Idle	Far	$\leq 10^{-9}$ A	≈1
S2_Overtaken	Moving	Far	$\geq 10^{-8}$ A	<1
S3_Stop	Idle	Mid	$\leq 10^{-9}$ A	≈1
S4_Fellow	Moving	Mid	$\geq 10^{-8}$ A	>1
S5_Accelerate	Idle	Near	$\leq 10^{-9}$ A	≈1
S6_Roll-Back	Moving	Near	$\geq 10^{-8}$ A	>1

References

[1] Y. Wang, H. Wu, L. Xu, H. Zhang, Y. Yang, Z. L. Wang, *Sci. Adv.* **2020**, *6*, eabb9083.
[2] S. Kumar, X. Wang, J. P. Strachan, Y. Yang, W. D. Lu, *Nat Rev Mater* **2022**, *7*, 575.
[3] M. Prezioso, M. R. Mahmoodi, F. M. Bayat, H. Nili, H. Kim, A. Vincent, D. B. Strukov, *Nat Commun* **2018**, *9*, 5311.
[4] W. Wang, G. Pedretti, V. Milo, R. Carboni, A. Calderoni, N. Ramaswamy, A. S. Spinelli, D. Ielmini, *Sci. Adv.* **2018**, *4*, eaat4752.
[5] Y. Li, J. Lu, D. Shang, Q. Liu, S. Wu, Z. Wu, X. Zhang, J. Yang, Z. Wang, H. Lv, M. Liu, *Advanced Materials* **2020**, *32*, 2003018.
[6] Y. Li, Z. Xuan, J. Lu, Z. Wang, X. Zhang, Z. Wu, Y. Wang, H. Xu, C. Dou, Y. Kang, Q. Liu, H. Lv, D. Shang, *Adv Funct Materials* **2021**, *31*, 2100042.
[7] S. Subbulakshmi Radhakrishnan, S. Chakrabarti, D. Sen, M. Das, T. F. Schranghamer, A. Sebastian, S. Das, *Advanced Materials* **2022**, *34*, 2202535.
[8] Y. Zhou, J. Fu, Z. Chen, F. Zhuge, Y. Wang, J. Yan, S. Ma, L. Xu, H. Yuan, M. Chan, X. Miao, Y. He, Y. Chai, *Nat Electron* **2023**, *6*, 870.
[9] S. Yang, D. Li, J. Feng, B. Gong, Q. Song, Y. Wang, Z. Yang, Y. Chen, Q. Chen, W. Huang, *Adv Elect Materials* **2024**, *10*, 2400075.
[10] L. Chen, S. Karilanova, S. Chaki, C. Wen, L. Wang, B. Winblad, S.-L. Zhang, A. Özçelikkale, Z.-B. Zhang, *Science* **2024**, *384*, 660.
[11] S. Subbulakshmi Radhakrishnan, A. Sebastian, A. Oberoi, S. Das, S. Das, *Nat Commun* **2021**, *12*, 2143.
